# Psychosis spectrum illnesses as disorders of prefrontal critical period plasticity

**DOI:** 10.1038/s41386-022-01451-w

**Published:** 2022-09-30

**Authors:** Sophia Vinogradov, Matthew V. Chafee, Erik Lee, Hirofumi Morishita

**Affiliations:** 1 Department of Psychiatry & Behavioral Science, University of Minnesota Medical School, Minneapolis, MN USA; 2 Department of Neuroscience, University of Minnesota Medical School, Minneapolis, MN USA; 3 Masonic Institute for the Developing Brain, University of Minnesota Medical School, Minneapolis, MN USA; 4 University of Minnesota Informatics Institute, University of Minnesota, Minneapolis, MN USA; 5 Department of Psychiatry, Neuroscience, & Ophthalmology, Icahn School of Medicine at Mount Sinai, New York, NY USA

**Keywords:** Synaptic plasticity, Medical research

## Abstract

Emerging research on neuroplasticity processes in psychosis spectrum illnesses—from the synaptic to the macrocircuit levels—fill key gaps in our models of pathophysiology and open up important treatment considerations. In this selective narrative review, we focus on three themes, emphasizing alterations in spike-timing dependent and Hebbian plasticity that occur during adolescence, the critical period for prefrontal system development: (1) Experience-dependent dysplasticity in psychosis emerges from activity decorrelation within neuronal ensembles. (2) Plasticity processes operate bidirectionally: deleterious environmental and experiential inputs shape microcircuits. (3) Dysregulated plasticity processes interact across levels of scale and time and include compensatory mechanisms that have pathogenic importance. We present evidence that—given the centrality of progressive dysplastic changes, especially in prefrontal cortex—pharmacologic or neuromodulatory interventions will need to be supplemented by corrective learning experiences for the brain if we are to help people living with these illnesses to fully thrive.

## Introduction

Over the years, many investigators have proposed that psychosis spectrum illnesses arise due to progressive changes in neural microcircuits that result in disturbances in experience-dependent plasticity and in the development of maladaptive cortical representational systems (e.g., [1–5]. While the concept is not new, emerging research on neuroplasticity processes—from the synaptic to the macrocircuit levels—fills key gaps in our models of pathophysiology and opens up important treatment considerations. Indeed, we will argue that these illnesses are best conceptualized as disorders of adolescent critical period prefrontal plasticity.

Of course, “brain plasticity” means everything and nothing, since changing in response to the environment is what the brain does. And it is impossible for one review article to even skim the surface of such a huge field of research. To start with, there are multiple types of interacting/overlapping neuroplasticity processes at multiple levels of scale. Experience-dependent (Hebbian) plasticity mechanisms at both the microcircuit and systems level need to be distinguished from spike-timing dependent, homeostatic, and metaplasticity operations, for example (Table 1). At the same time, it is arbitrary to separate these processes, since they all reverberate with each other and with intrinsic neuronal excitability and neuromodulatory input to coordinate—or stabilize against—changes in network connectivity and function that occur as the brain interacts with its environment. Changes in one of these processes affects the others, and all of these mechanisms are relevant to psychosis.

**Table 1:** Summary of key neuroplasticity processes and examples of their disruption in psychosis spectrum illnesses.

Key neuroplasticity processes	Definition	Examples of disruption in psychosis spectrum illnesses
Synaptic plasticity	The activity-dependent modification of the efficiency or strength of synaptic transmission at pre-existing synapses	Abnormally elevated microglial synaptic engulfment [138]
Spike-timing dependent plasticity	The process by which synaptic transmission is enhanced or depressed, based on the order and precise temporal interval between presynaptic and postsynaptic spikes; its relative importance varies across synapses and activity regimes	Spike timing activity in PFC is disrupted by NMDAR blockade in a nonhuman primate model of schizophrenia [17]
Critical period (experience-expectant) plasticity	An epoch in brain development where large-scale changes in neuronal response selectivity can be induced by passive exposure to specific forms of environmental inputs; requires that intrinsic maturational programs and environmental inputs are temporally aligned	Alterations in critical period transcriptomics [145].
Hebbian (experience-dependent or activity-dependent) plasticity	A form of synaptic plasticity which is induced by and further amplifies correlations in neuronal activity; outside of the critical period, it is harnessed as a function of alertness, attention, incentive salience of inputs, and behavioral outcome	–Impaired LTP-like responses in visual cortex [191]; –Impaired neurostimulation-induced LTP- and LTD-like effects [169]
Homeostatic plasticity	A set of neuronal changes that restores activity to a setpoint following perturbation; creates balance between intrinsic excitability and synaptic strength, and between network excitation and inhibition; coordinates changes in circuit connectivity	Hypothesized functional and structural and synaptic downregulation that occurs in response to initial microcircuit hyperexcitation [4]
Compensatory plasticity	The capacity to adapt to the loss of neural macrocircuit (or cortical sector) function by undergoing plastic changes in neural interactions, circuit structure, and/or circuit connectivity.	–Thalamic hypoconnectivity between prefrontal-striatal-cerebellar regions and hyperconnectivity with sensory-motor cortices [177] –Abnormal recruitment of VWFA during auditory working memory in people with psychosis [181]
Synaptic scaling	A negative feedback mechanism to changes in the level of network activity, in which the synaptic strengths of a neuron are modified by regulating synaptic receptors following a scaling factor. The total synaptic input is adjusted to match the neuron’s homeostatic range while preserving the relative differences among synaptic weights	Computational modeling of EEG and fMRI data indicating reduced pyramidal cell synaptic gain [192]
Metaplasticity	Activity-dependent changes in neural functions that modulate subsequent synaptic plasticity responses.	Early childhood adverse experiences increase the risk for schizophrenia [51]

In this selective narrative review, we focus on recent research findings related to three themes, emphasizing alterations in spike-timing dependent and Hebbian plasticity that occur during prefrontal system development:

1. Experience-dependent dysplasticity in psychosis emerges from activity decorrelation within neuronal ensembles.
2. Plasticity processes operate bidirectionally: Deleterious environmental and experiential inputs shape microcircuits.
3. Dysregulated plasticity processes interact across levels of scale and time and include compensatory mechanisms that have pathogenic importance.

The evidence supporting each of these themes is indirect and relies on basic science experiments and computational modeling—we cannot directly assess microcircuit function in humans, while macrocircuit plasticity can only be inferred from neuroimaging evidence and TMS studies. However, each of these themes helps to integrate a disparate range of research findings while broadening our view of what it will take to develop effective restorative, preventive, and perhaps even curative interventions for individuals who are vulnerable to persistent psychosis. We present evidence that—given the centrality of progressive dysplastic changes, especially in prefrontal cortex—pharmacologic or neuromodulatory interventions will need to be supplemented by corrective learning experiences for the brain if we are to support individuals living with these illnesses to fully thrive.

## Experience-dependent dysplasticity in psychosis emerges from activity decorrelation within neuronal ensembles

**Fig. 1:**
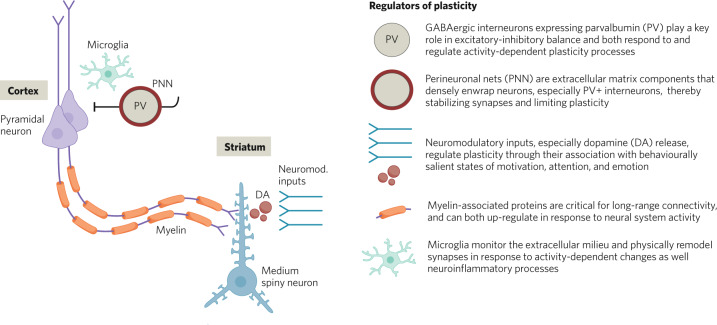
Plasticity regulators in prefrontal microcircuits. Simplified diagram of prefrontal microcircuit components and key regulators of plasticity. (Adapted with permission from ref. [142]).

Dysplasticity mechanisms are often posited to originate from primary alterations in plasticity-relevant genes and/or gene expression [6], but they also manifest as downstream consequences of many other factors that affect microcircuit function and the coordinated activity within neuronal ensembles. This includes factors that affect excitation-inhibition balance within cortical circuits, such as glutamatergic and GABA-ergic signaling; dopamine modulation of cortical and subcortical systems; the integrity of perineuronal nets; and microglial activity in synaptic remodeling (see Fig. 1).

### Different pathogenic models converge on decorrelation of neuronal ensemble activity

Hamm et al. ([7]) studied two distinctly different mouse models relevant to schizophrenia: blockade of NMDA receptors (NMDAR) via chronic ketamine and 22q11.2 microdeletions. The two models diverged in key neurophysiological indices (such as background gamma oscillations and overall levels of activity), but they *converged* at the neuronal ensemble level, both showing weaker neuronal coactivity patterns (attractor states) in the visual cortex at rest and during visual stimulation. These two different manipulations relevant to schizophrenia showed decorrelated neuronal ensemble firing rate patterns over time, such that fewer and shallower attractor states were visited by the network. Zick and Chafee studied NMDAR blockade in monkeys and a 22q11.2 microdeletion model in mice, and found that both manipulations showed decorrelated activity in prefrontal networks at a finer time scale, on the order of milliseconds, reducing the frequency of synchronous 0-lag spikes observed between pairs of simultaneously recorded prefrontal neurons [8]. Such a loss of synchronous spiking could disconnect prefrontal circuits via spike-timing dependent plasticity mechanisms. Consistent with that possibility, less information was transmitted at monosynaptic lags between prefrontal neurons in both the monkey drug and mouse genetic models [8].

Disrupted attractor states and disrupted spike timing may be considered two forms of temporal decorrelation of neuronal activity unfolding at different time scales. Attractor states, defined as stable patterns of firing rates over neuronal ensembles, capture covariation in average firing rate between neurons at behavioral time scales—e.g. over hundreds of milliseconds as sensory, cognitive, and motor processes wax and wane, or as networks revisit stable activity patterns in spontaneous activity. Spike synchrony also reflects temporal covariation in neuronal activity (spiking), but at a millisecond time scale. Although clearly distinct in terms of their underlying causes and impacts on synaptic and system plasticity, both are forms of activity timing and correlation that are likely disrupted in psychosis spectrum illnesses. Disruptions in activity timing are evident at larger spatial scales as well. For example, local field potentials exhibit more frequent spike-wave events at the cortical level and weaker inter-hippocampus synchrony in rats with neonatal ventral hippocampus lesions, a neurodevelopmental model of schizophrenia [9].

Thus, it appears that various biological mechanisms relevant to psychosis spectrum illnesses converge to alter the the ability of neurons to form or sustain complex coordinated patterns of activity at multiple levels of temporal and spatial scale: from spike timing in pairs of neurons, to the coordinated activation of neuronal ensembles to form stable attractor states, to the orchestration of activity within networks. Some of these timing anomalies may derive from alterations in the precise balance between glutamatergic excitatory inputs relative to GABA-ergic inhibitory inputs in local circuits, referred to as E-I balance. The maintenance of a precise E-I balance appears critical to promote the optimal temporal order of synaptic action potentials necessary for adaptive spike-timing dependent plasticity; however, as O’Donnell et al. point out [10], a simple one-dimensional E-I balance model does not fully account for the multidimensional computational functions of neural microcircuits (including how they are modified over development and how changes in certain cellular subcomponents have more of an influence on circuit function than others).

Nonetheless, cortical plasticity deficits can arise by both enhancing excitation and by reducing inhibition in microcircuits [11]. Specifically, disruption of the E-I balance in favor of excitation leads to impaired cortical plasticity in vivo [12]. This disruption can happen as a consequence of both (1) deletion of the NMDAR subunit Grin2a gene, a genetic risk for schizophrenia [13, 14], which enhances excitation by prolonging glutamatergic synaptic responses; (2) reducing GAD65, a key enzyme needed to generate synaptic GABA, which lowers cortical inhibitory tone. Both deficits can be rescued by enhancing cortical inhibition via diazepam, an allosteric modulator of the GABA-A receptor [12]. Similarly, Cisneros-Franco et al. have shown that destabilization of sensory representations in the aging rat brain is associated with decreased GABA concentration, a reduction in parvalbumin perineuronal nets, and decreased cortical inhibition, which in turn are related to dysregulated (overly active but unstable) plasticity; these processes can be reversed by increasing inhibitory tone via diazepam [15]. This example illustrates how degraded sensory inputs–in this case, due to aging—can affect E-I balance and result in abnormal plasticity operations (discussed further in Section “Plasticity processes are bidirectional: Deleterious environmental and experiential inputs shape macro- and micro-circuit function”).

### Decorrelation of neuronal ensemble activity leads to the unwiring of local circuits

Subtle abnormalities in E-I balance (via impaired glutamatergic or interneuron function, for example), with resultant spike-timing disruptions and decorrelated neuronal activity, will end up disrupting synaptic connectivity in local prefrontal circuits via Hebbian principles [16, 17]—“neurons that don’t fire together won’t wire together”. This in turn impairs the engagement of plasticity regulators and the adaptive tuning of neuronal ensemble representational processes. In other words, as cells stop firing together and local circuits become less coordinated, cortical attractor states become destabilized and the information they encode is degraded. In some individuals this process might become accelerated during the normal prefrontal synaptic pruning that occurs during adolescence—the critical period for the maturation of executive and other higher-order socio/cognitive functions in humans [18]. Other contributing factors could include genetically-determined *excessive* synaptic pruning mechanisms that may occur in some forms of schizophrenia [19] or disruptions of in utero brain development [20, 21] that result in impaired prefrontal circuit function early in life.

**Fig. 2:**
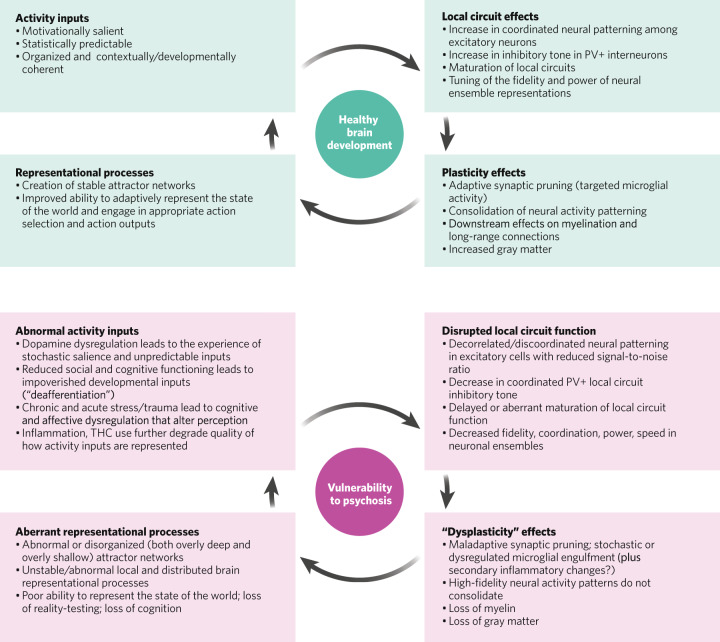
Neuroplasticity processes in healthy development and in vulnerability to psychosis. Hypothesized assocations linking neural activity inputs, microcircuit function, plasticity effects, and representational processes—as they occur in healthy development and are disrupted in individuals who are vulnerable to psychosis.

In any case, as prefrontal local circuits gradually unwire, adaptive excitatory interactions between neurons may eventually fall below the physiological threshold needed to meaningfully sustain coordinated activity within ensembles. At this point, further activity-dependent disconnection and further pruning could start to drive one another in a mutually reinforcing but pathogenic feedback cycle [8], since microglia sculpt neuronal ensembles as a function of their activity inputs (less coordinated activity input = more pruning) [22] and spike timing synchrony depends on intact recurrent local networks that would be getting weaker as pruning progresses. Compensatory reductions in GABAergic inhibitory tone may occur as a result [23], which further impairs the ability of neuronal ensembles to form or sustain the complex patterns of activity needed for adaptive behavior, and which creates the conditions for dysregulated plasticity to begin operating (as we discuss further in Section “Dysregulated experience-dependent plasticity processes interact across levels of scale and time and include compensatory mechanisms that have pathogenic importance”). This is a reversal of the adaptive plasticity that normally occur during healthy critical periods and healthy development, where statistically reliable inputs to well-functioning prefrontal local curcuits engage enhanced E-I coupling and coordinated neuronal activity patterns, resulting in the maturation of inhibitory interneurons and the perineuronal nets which surround them, increases in gray and white matter, and improved adaptive representational processes (see Fig. 2). (Critical period plasticity refers to specific epochs during development when cortical representations are passively organized in response to statistically predictable inputs from the environment in order to consolidate perceptual and cognitive skills critical for survival. Classically, this has referred to periods during which sensory, motor, and language abilities are acquired. More recently, the role of prefrontal plasticity in adolescence and the acquisition of higher-order executive and social skills is recognized. Plasticity continues throughout adulthood but becomes tightly regulated and depends increasingly on attentional and motivational state, behavioral practice, and behavioral outcome.)

### Dopamine function/dysfunction amplifies the effects of local circuit pathology

Near-synchronous spiking of pre- and postsynaptic local circuit neurons is required to establish a persistent eligibility trace within dendritic spines that renders specific synapses susceptible to potentiation by cortical dopamine [24, 25]. This suggests that the synaptic specificity of dopamine relies on the spike timing history of individual synapses; loss of synchronous spiking in prefrontal local circuits at the millisecond time scale would be expected to degrade the mechanism that confers synapse specificity on dopamine-mediated signals and lead to indiscriminate synaptic potentiation and the further disorganization or aberrant patterning of neuronal ensembles in the prefrontal cortex [26]. As is well established, striatal presynaptic dopamine synthesis, storage, and release is increased in psychosis spectrum illnesses [27–31], while cortical dopamine release is reduced, and the two appear to be inversely correlated [32–34]. The fact that NMDAR synaptic deficits [35–37] and dopamine disruptions are both contributors to illness expression indicates that the illness not only affects the number of dendritic spines [38, 39], but likely changes how dendritic spines work as biochemical machines to respond to dopamine inputs and to adjust the strengths of synapses to spiking patterns in networks—i.e., to engage in adaptive plasticity [16].

The emergence of psychosis, which usually occurs during mid-late adolescence, is heralded by an increase in dopamine signaling in the dorsal associative striatum [28, 40, 41]. The degree of excessive dopamine synthesis predicts conversion to psychosis and correlates with positive symptom severity [42]. Dorsal striatum receives direct projections from the dorsolateral prefrontal cortex [43] and provides feedback to it via relays in ventral anterior and medio-dorsal thalamic nuclei [44]. By virtue of this cortico-striatal-thalamo-cortical loop, a disturbance in dopamine modulation in the dorsal striatum will propagate throughout the network to disrupt neural ensemble dynamics in the dorsolateral prefrontal cortex, affecting its capacity to sustain internal representations of external stimuli stored in working memory and to engage in efficient predictive coding [45–47]. Behaviorally, striatal dopamine plays a key role in tagging reward prediction errors (the discrepancy between expected and actual rewards) and in assigning salience to environmental stimuli, including threat, novelty/surprise and motivational importance. The interaction of these functions with a faulty cortical processor could result in the “burning in” of aberrant attractor networks corresponding to false perceptions or aberrant interpretations as a secondary consequence of abnormal learning while the brain is in an unstable or dysplastic state [27, 48].

Though striatal hyperdopaminergia plays a fundamental role in the emergence of frank psychosis, active debate remains on the exact nature and direction of the relationship between striatal dopamine, cortical dopamine, and models of cortical dysfunction in schizophrenia [49]. For instance, in an experimental manipulation in mice that produced elevated synaptic pruning and cortical ensemble dysfunction, striatal hyperdopaminergia and psychosis-relevant behaviors were secondarily generated that were subsequently reduced by antipsychotic medication [50]. Cortical synaptic dysfunction can thus precede and induce striatal hyperdopaminergia, though the exact mechanisms are unknown. Hyperresponsivity in dopamine neuromodulation due to prior trauma exposure can also be a critical factor that tips the system beyond its homeostatic capacities in individuals who are vulnerable to high-stress conditions [51].

### Local circuit pathology propagates up the information processing hierarchy

Local neuronal ensemble coordination, in conjunction with neuromodulatory input that is appropriate to the behavioral demands at hand, is the *sine qua non* of adaptive experience-dependent plasticity at the higher systems level [52]. Cortical networks at higher association levels are integrators that operate with dynamic (and relatively brief) time constants, and plasticity-induced changes in cortical representations are dependent on the integration of coincident inputs. A range of processes—including membrane potential, noise, and input firing rate—can modify the sensitivity of cortical neurons to the degree of temporal correlation between their synaptic inputs [53]. Thus, the stronger the selectivity, reliability, and coordination of the neurons in lower levels of the network that feed coincident inputs into prefrontal neurons, the stronger is the power of those inputs to drive adaptive plastic remodeling across the distributed network. Moreover, finely tuned coordination of activity is what enables higher-order association regions to sustain the reverberant activations needed to accomplish complex cognitions and behaviors.

Lower-level disruptions in the ability of neuronal ensembles to sustain complex patterns of activity that accurately represent the state of the world, and/or inappropriately scaled neuromodulation of that activity, thus end up propagating distortions that affect the fidelity of higher-level prefrontal circuit computations. As Yang et al. have shown, a global microcircuit E-I imbalance will preferentially affect long-range prefrontal connectivity patterns, due to the fact that there are physiological differences (stronger recurrent excitation) in association vs. nonassociation cortices [54]. This includes the representation and meaning of perceptual information as well as associated predictive, categorization, memory, and action-output information—distortions which are posited to give rise to subjective psychosis symptoms. As the world becomes characterized by unpredictable aberrant percepts and experiences that appear salient, a range of representational patterns may be shifted into abnormal configurations which are then learned into place. Note that during critical period plasticity, the cortex learns to represent the experiences it is being exposed to as long as they show statistical regularities—these activity patterns induce the tuning of neuronal ensembles and circuit maturation. In a state of dysregulated plasticity during the first episode of psychosis, symptoms might arise from sudden transitions to abnormal neural representation patterns, and subsequent plastic changes then lock that new neural firing pattern in as an attractor state [55, 56].

### Plasticity-informed interventions can aim to target cortical circuit dysfunction

If discoordinated local neuronal ensemble dynamics is a key common pathogenic feature in psychosis spectrum illnesses that lead to system-level dysplasticity during a critical period of prefrontal cortical development, then it follows that treatments which target abnormal neural coordination should be useful in psychosis. What is the evidence thus far?

#### Assessing abnormal neuronl ensemble coordination: a gateway to treatment development

Since there is no direct way to measure neuronal ensemble decorrelation in the human brain in order to determine its responsiveness to interventions, clinical researchers must resort to indirect methods. Neurophysiological indices of the fast temporal integration and resolution of auditory inputs, such as the 40 Hz gamma-band auditory steady state response (ASSR) and the auditory mismatch negativity response (MMN), are dependent on intact NMDAR function and probably serve as indirect proxies of local neural ensemble coordination in auditory cortex and also across thalamo-cortical circuit integration (they also index other relevant processes, such as short-term hypoplasticity, which are themselves likely to be manifestations of local circuit dysfunction). Deficits in ASSR and MMN are observed in psychosis spectrum illnesses, predict conversion to psychosis in high-risk individuals, and are noted to be key biomarkers for new drug development. Psychosis-relevant deficits in these two measures can be induced by NMDAR antagonists in humans and in rodent and monkey models [57–59].

In a recent large-N structural equation modeling study, ASSR and MMN deficits were found to significantly contribute to unique hierarchical “bottom-up” effects on neurocognition, symptoms, and functioning in patients [60], consistent with the notion that changes in local circuit dynamics that affect lower level perceptual processing have widespread deleterious behavioral consequences as their effects propagate up the information processing hierarchy. This same group of investigators determined that measures of early auditory information processing, particularly MMN, had a direct effect on general (not modality-specific) cognition (*p* < 0.001), that cognition had a direct effect on negative symptoms (*p* < 0.001), and that both cognition (*p* < 0.001) and negative symptoms (*p* < 0.001) had direct effects on functional outcome.

This model further predicted that a treatment that provided a 1 µV improvement in the MMN response would result in improvements in cognition with an effect size of d = 0.78 and improvements in psychosocial functioning with an effect size of d = 0.28 [61]. Given the dysplasticity processes described earlier, it is unlikely that a short-term improvement of 1 µV in the MMN response will *immediately* lead to more adaptive higher-order cognitions and functioning—rather, improvements in lower-order information processing would need to be initiated, sustained, and combined with treatments that shift pre-existing maladaptive learned behavior. Coordinated neuronal ensembles are just one step in the translation of experience into the brain and generating adaptive behaviors: pulling on the molecular levers that affect these ensembles likely needs to be supplemented by methods that generate and hardwire corrective macrocircuit patterns as well.

#### Glycine and D-serine

Glycine and d-serine are co-agonists at the NMDAR and in a range of studies have been shown to enhance NMDAR functioning within microcircuits. They also reverse the abnormal MMN response [62] and even though they are not yet an actionable treatment path, emerging data in schizophrenia are encouraging [63, 64]. Moreover the molecular actions of these co-agonists suggest exciting avenues for multimodal treatment approaches. Kantrowitz et al. combined higher-dose d-serine (60 mg/kg) with two sessions of auditory tone-matching training in individuals with schizophrenia and demonstrated both a significant improvement in auditory MMN and in task performance (*d* = 0.7), providing evidence of NMDAR target engagement by d-serine as well as adaptive training-induced neuroplasticity [65]. Well-powered studies combining d-serine with cognitive training are a next important step, especially given very recent findings that d-serine is required for the fine-tuning of glutamatergic neurotransmission, neuronal excitability, and synaptic plasticity in the prefrontal cortex through the actions of dopamine at D_1_ and D_3_ receptors [66]. D-serine could be a powerful permissive agent that—when combined with appropriate cognitive and behavioral interventions—exerts its effects by enhancing microcircuit functioning and inducing adaptive experience-dependent synaptic plasticity across distributed systems.

#### Neuroplasticity-informed cognitive training

Neuroplasticity-informed cognitive training methods apply principles derived from several decades of research on learning-induced plasticity in animal experiments to the design of human interventions. As an example of two experiments which are relevant to psychosis spectrum illnesses, De Villers-Sidani et al. studied cognitive and functional brain changes in aged rats and in young adult rats exposed to prolonged broadband noise [67, 68]. They showed that, with the degraded fidelity of auditory processing that occurs in aging, neuronal ensembles in primary auditory cortex had reduced firing synchrony, reduced cortico-cortical interactions, and a weak relative suppression to repetitive, high-probability background sounds, resulting in a loss of salience of novel, low-probability stimuli (similar to the abnormal MMN seen in chronic psychosis). The aged cortex also showed reduced parvalbumin (PV) staining of inhibitory interneurons, reduced PV cell dendritic arborization, and a significant decrease in myelin basic protein, consistent with a homeostatic response to decreased and less reliable sensory inputs that occurs with aging. Similar changes were found in young adult rats exposed to the degraded auditory representations from prolonged broadband noise (Table 2).

**Table 2:** Brain dysplasticity changes observed in aged rats with age-related degradation in sensory perception and in young rats exposed to prolonged broadband noise; analogous observations in psychosis; and the reversibility of these changes in the rat model after perceptual training.

Brain dysplasticity changes observed in aged rats and in young rats exposed to prolonged broadband noise [15, 67, 68]	Parallel findings in psychosis spectrum illness	Degree to which changes are reversible by intensive auditory discrimination training in the animal model [68]
Degraded reliability of temporal coding Decreased cortical firing synchrony	(Inferred) Decreased amplitude and synchronization of the 40-Hz ASSR response [59]	++
Decreased cortico-cortical interaction	Consistent with the “disconnection model” [193]	++
Decreased relative responses to rare stimuli	Impaired ERP responses during oddball tasks Reduced MMN response [62]	++
–Decreased PV staining; weak PV expression in PV + neuron –Simplification of PV + cell dendritic arborization	Reduced PV interneuron density seen in post-mortem samples [148]	+
Decreased myelin basic protein density in the cortex	Decreased superficial white matter [194]	+
Poorer auditory frequency discrimination	Impaired auditory feature discrimination [195]	+
Increased false-positive errors during behavior	Consistent with increased false-alarm responses on memory tasks [196]	+

In the rat model, exposing the brain to degraded neural activity inputs—whether from aging or from exposure to broadband noise—drives a range of physiologic changes in cortex that are consistent with dysregulated plasticity responses (as if the cortex has been driven into an immature state). Analogous physiologic findings are seen in psychosis spectrum illness. In the rat model, these physiological changes are reversible in response to perceptual training that improves the fidelity and power of cortical sensory representations.

After intensive training on an auditory oddball discrimination task, the aged rats showed a nearly complete reversal of the majority of these functional and structural cortical impairments, along with improved frequency discrimination and reduced false-positive errors [68]. These experiments highlight similarities among the dysplastic cortical changes observed in two animal models– aging and exposure to perceptual noise– and what is seen in psychosis, which we discuss in more detail further below. It also demonstrates that these dysplastic changes are reversible after a well-defined course of specifically targeted, behaviorally-relevant, sustained and patterned neural activity (see Table 2).

In the human application, individually adaptive behavioral exercises are delivered to evoke high-fidelity perceptual processing at speed, which requires increases in the coordination and amplitude of neural responses at the level of both sensory and prefrontal cortices [69]. Exercises require close attentional control and operate at an 80% correct threshold, yielding a dense reward schedule (that presumably engages dopaminergic tagging) and driving individuals to train at their response threshold. Exercises are designed to improve the selectivity of neural responses to specific auditory or visual stimuli; the resolution of spectrotemporal or spatiotemporal stimulus complexity; the representation of stimulus magnitudes and modulation rates; successive-signal segmentation and integration; stimulus duration and inter-stimulus interval resolution and estimation; stimulus sequencing; stimulus source location or identification; signal-to-noise conditions for stimulus representation; and response reliability. The goal is to drive more strongly coordinated populations of neurons that efficiently represent and act upon salient auditory or visual information. In order to drive and consolidate the needed cortical plastic changes in individuals with impaired cognition, exercises must be delivered at an effortful level (~20% error rate), on a sufficiently intensive schedule (3–5 times per week), and for a sufficient duration of training (20–50 hours). Otherwise, little cognitive benefit is seen.

In studies of auditory system training in people with psychosis spectrum illnesses, abnormal baseline ASSR and MMN normalized after one hour of training exposure [70]. The presence of an abnormal baseline ASSR and MMN also predicted the overall general cognitive improvement seen after many weeks of training (20–50 hours), though interestingly, the full course of training did not induce lasting changes in ASSR or MMN [70, 71] (Both studies had small Ns and the dynamic picture of MMN change over time in response to distributed system perturbations is complex, possibly due to the presence of compensatory neural system changes in psychosis, as seen in neuromodulation studies [64]).

These findings suggest that psychosis-spectrum individuals with neurophysiological indices suggestive of neural discoordination in early auditory processing (abnormal ASSR or MMN) may be especially responsive to this form of auditory cognitive training. Imaging work has demonstrated that this “lower-level” training results in extensive plastic changes in task-related activations within auditory cortex as well as higher-order prefrontal cortical systems; improvement in a corollary discharge measure; and preservation of thalamic volume in early phase individuals [72–76]. Whether this translates into adaptive changes in local neuronal ensemble synchrony is unknown, though may be presumed to occur given the increased amplitude observed in M100 auditory responses in primary auditory cortex [76]. Finally, this form of plasticity-informed training in early psychosis is associated not just with improved general cognition but also with reduced psychosis symptoms at 6 month follow-up, consistent with the idea that lower level corrective plastic changes can “propagate up” the information processing hierarchy to improve higher order cortical representations and subjective experience [77].

There is very indirect and tentative evidence of the interaction between this form of intensive cognitive training and the NMDAR system. Intensive cognitive activity in animals increases brain levels of d-serine, and serum levels of d-serine are known to be lower in people with schizophrenia compared to healthy controls. [78] showed that, after auditory system training in people with psychosis, there was an increase in serum d-serine that was positively correlated with cognitive gains, suggesting that endogenous d-serine—and hence NMDAR function—is involved in the plasticity processes induced by training. This form of cognitive training has also been associated with significant increases in serum BDNF, again providing tentative evidence that cognitive training induces molecular/cellular effects consistent with neuroplasticity [79].

#### Into the future: Therapeutic optogenetic reprogramming of neuronal ensembles

Finally, while the idea is in the realm of science fiction, it may be possible in the not-too-distant future to selectively activate and imprint specific cortical neuronal ensembles using optogenetic techniques [80], which in theory could allow for the reprogramming of aberrant cortical circuit function, the restabilization of an abnormal attractor landscape, and reversal of psychosis symptoms [7, 81] used two-photon optogenetics to repetitively activate defined neural populations in the visual cortex of mice; this intervention generated (imprinted) neuronal ensembles that then recurred spontaneously and remained coactive on consecutive days. These results suggest that it may be possible to not only deliberately enhance or create cognitively adaptive neuronal ensembles and attractor networks in cortex, but also to deliberately deactivate or reconfigure maladaptive neuronal ensembles, in order to to support recovery and functioning.

## Plasticity processes are bidirectional: deleterious environmental and experiential inputs shape macro- and micro-circuit function

While many have proposed a causal chain linking cellular dysfunction to microcircuit impairment to progressive maladaptive experience-dependent plasticity, we emphasize that plasticity mechanisms can and do operate bidirectionally [82]. The brain evolved so that behavior and experience could shape (and re-shape) neural activity, and neural activity modulates the growth and sculpting of dendritic spines by inducing a range of changes in gene transcription and protein synthesis, including changes related to microglial and cytokine activation; these changes in turn drive modifications at the local circuit, neuronal ensemble, and systems level [26, 83–85].

### Deleterious experiences affect brain microcircuit function

Deleterious experience-induced changes in distributed neural network functioning—including changes in the neuromodulatory, endocrine, and immune systems—can lead to changes in microcircuit physiology and cellular firing patterns [86–88]). Each individual’s prior and current social and environmental exposures, along with their emotional and behavioral responses, affect the circuitry underlying sensory, cognitive, and affective processes and can push the circuitry in the direction of adaptive or pathological patterns. As an example, extremely deleterious mental/emotional states—such as those induced by repeated severe psychological/physical trauma or prolonged sleep deprivation—can drive hyperarousal and persistent aberrant assignment of salience that then contribute to psychosis through enduring macrocircuit dysfunction and epigenetic changes that alter gene expression and synaptic function [89–92]. This has been especially well studied in the stress literature, but is relevant across all significant human-environment interactions.

### Adolescence is a critical period for plasticity in executive, social, and functional capacities

Adolescence is the key critical period for experience-dependent plasticity in executive, social, and adult functional capacities, where exposure to (and learning from) appropriate stimuli in these domains must occur in order to prepare the individual for adaptive adult behavior and future successful social learning. An individual who is in the prodrome for psychosis or who is moving through the first few episodes is undergoing highly disruptive and stressful perceptual, cognitive, and emotional experiences while also experiencing social and functional failures and decline. People who are at high risk for, and who enter into, their first episodes of psychosis, have a higher history of being bullied and experience impoverished and unsatisfactory social interactions (e.g., [93, 94]). Individuals with early psychosis view themselves as being of lower social rank and inferior in relation to matched controls, report engaging in submissive behaviors more frequently, and feel more entrapped by external events [95].

Let us focus for a moment on this social dysfunction. Recent research in rodents indicates unequivocally that adolescence is a “sensitive period” for experience-dependent plasticity in social behavior [96]. For example, two weeks of juvenile isolation leads to abnormal adult sociability and a failure to activate medial prefrontal cortex neurons that project to the posterior paraventricular thalamus (mPFC→pPVT) during social exposure in adulthood. Both reduced excitability of mPFC→pPVT neurons and increased inhibitory input drive from somatostatin interneurons are observed, indicating fundamental changes in the circuit mechanism underlying the sociability deficits [97]. In humans, adolescence is considered a critical plasticity period for the acquisition of lifelong social and functional skills, and cognitive science has established that the mPFC is a central node in the network supporting representation of self and other, social reward, and social interactions. Social reward salience is diminished in psychosis, and may explain aspects of defeatist beliefs and reduced motivation.

Given this, we can view the progressive social and functional impairment that occurs during adolescence and early adulthood in more severe or persistent forms of the illness as a *social deafferentation* syndrome [98]—a pathogenic mechanism in and of itself that will, through cumulative maladaptive plastic changes during a sensitive period, substantially affect maturation and functioning within prefrontal neural circuits. Consistent with this idea, both childhood maltreatment and solitary confinement are associated with the emergence of psychosis; and adoptees at high genetic risk for psychosis are protected by a healthy rearing-family environment but show psychiatric problems when reared in dysfunctional families [99]. At the very least, without the appropriate successful learning experiences, the trajectory of skills acquisition that needs to occur during the critical period of adolescence will be derailed, resulting in severe psychosocial impairment.

### Plasticity-informed interventions can harness corrective environmental and experiential forces

Successful treatment in the early phases of psychosis requires not just medications to reduce striatal hyperdopaminergia, but keeping the individual engaged with family, friends, school, and work. A neuroplasticity-based perspective conceptualizes these activities as capitalizing on the critical period for the acquisition of social skills and functional autonomy and as preventing further maladaptive plastic changes in the brain’s cognitive and socio-affective capacities for participating in society. Relationship with peers is especially important and has been found to predict clinical recovery, though it is not always explicitly addressed in treatment programs [100].

#### A neuroplasticity perspective on psychotherapeutic and psychosocial treatments

Current successful psychotherapies for psychosis spectrum illnesses teach stress-management skills and/or modify the beliefs, behaviors, and sense of self the individual forms around symptoms, especially during the critical developmental period for identity formation. Interventions focused on resilience, positive psychology, and social engagement all have a significant effect on the course of illness, self-stigma, and disability [101–104]. Evidence-based supportive employment programs enhance quality of life and appear to also confer clinical benefit [105].

From a neuroplasticity-based view, these treatments explicitly encourage the development of compensatory strengths-based cognitions, affects, behaviors, and sense of self that reduce stress and fear responses and that create new distributed macrocircuit configurations to competitively interfere with the aberrant perceptual and cognitive representations induced by psychosis. An especially important issue is when an individual’s self-representation becomes fused with the experience of psychosis—what we might call “lack of insight.” In the highly creative AVATAR therapy, patients select virtual avatars to represent their persecutory and derogatory hallucinations, and then engage in a progressive positive dialogue with the avatars via a therapist, to slowly modulate the intensity, negative valence, and significance of the hallucinations and “unfuse” from the aberrant perceptual experience [106].

Animal experiments show unequivocally that enriched environments increase adaptive plasticity and improve neuronal response properties, thus increasing the capacity for ongoing learning. Likewise, psychotherapies that harness corrective environmental and experiential forces induce adaptive systems-level plastic changes that are enduring. CBT for psychosis strengthens the neural connectivity between higher-order cognitive systems and those involved in threat and salience, potentially facilitating reappraisal, and these effects endure at 6 months [107]. Cortical reorganization that occurs after a course of multimodal psychological therapy predicts the subsequent recovery path of people with psychosis across 8 years [108]. Two years of cognitive enhancement therapy (CET), combining cognitive remediation with social skills group training, drove increased right DLPFC activity 2 years later that was associated with improved cognition, as well as increased functional connectivity between DLPFC and amygdala that was associated with improved emotion management [109, 110].

#### Using social cognitive training to enhance reward responsivity and social functioning

Impaired social cognition is a hallmark feature of many forms of psychosis spectrum illness and is a critical determinant of motivated behavior and functioning [111, 112]. A recent longitudinal causal discovery model in a large sample of first-episode individuals showed that baseline social-emotional ability was a direct causal factor for motivated behavior and in turn related to 6-month outcomes for social and occupational functioning [113].

Impaired basic social cognition capacities—such as emotion recognition, eye-gaze detection, and auditory emotional prosody discrimination—are highly responsive to neuroplasticity-informed targeted cognitive training, as shown in a recent double-blind randomized multi-site trial [114]. In secondary analyses, improvements in social functioning, virtual functional capacity, and motivation are also observed. Another double-blind RCT examined the benefit of adding social cognitive training to a neurocognitive training regimen and showed that the combination resulted in improved measures of emotional prosody and in self-reported reward responsivity [115]—improvements which increased over the course of training, were present at 6-month follow up, and were positively associated with improved social functioning. Unpublished data reveal that the group who received this combined training show improved accuracy on a monetary incentive delay task along with enhanced mPFC activation during reward anticipation; mPFC enhancement was positively associated with self-reported gains in reward responsivity (Subramaniam, manuscript in preparation). These findings are consistent with Subramaniam et al. [74], where combined social cognitive+neurocognitive training also enhanced mPFC activation during a self-other source memory task in a manner that correlated with improved social functioning 6 months later.

The PRIME app (Personalized Real-time Intervention for Motivational Enhancement) is another creative approach to improving motivated behavior and social functioning in first episode individuals [116]. Based on intrinsic motivation theory [117], the app is based on user-centered design principles and creates an on-line social community of young people experiencing a first episode of psychosis; it provides them with remote coaching and goal-setting as well as the opportunity to share their experiences and display their accomplishments to a community of peers. In a randomized trial, and as compared to the waitlist condition, people participating in PRIME had significantly greater improvements in self-reported depression, defeatist beliefs, self-efficacy, and motivation/pleasure, with improvements being maintained 3 months after the end of the intervention. Individuals in the PRIME condition also showed significantly greater improvements in social motivation (anticipated pleasure and effort expenditure) in a laboratory-based social trust game.

#### Into the future

The field is ready for a systematic neuroscience-informed treatment approach that is delivered as early as possible (for pioneering work with this concept, see refs. [118, 119]) and that focuses on corrective social and executive learning experiences for the adolescent prefrontal cortex within a multimodal context:

- NMDAR-enhancing medication to improve microcircuit functioning and create the conditions for optimal learning.
- Non-invasive neuromodulation to prime prefrontal circuits before or during cognitive training (see ref. [120]).
- A course of well-defined cognitive and social cognitive training to drive adaptive plastic changes in brain circuits that support fundamental representational capacities.
- Evidence-based and user-centered digital tools to support involvement with peers, enhance motivation, and promote engagement with treatment and recovery.
- Evidence-based psychotherapeutic and psychosocial programs to provide higher-level corrective skills, strategies, and experiences. A special focus on social skills training—which is very effective but not yet systematically implemented—will be important, as will interventions to promote successful peer activities and romantic relationships as part of essential critical period experiences.

For a complementary neurophysiologic intervention, Yamamuro et al.—based on their findings of the dysplastic effects in adults of “social deafferentiation” during the critical period in adolescent mice—proposed that chemogenetic or optogenetic stimulation of mPFC→pPVT neurons in adulthood paired with social interaction could rescue the dysplastic social network, restore its function, and reverse the sociability deficits caused by juvenile isolation. Similar macrocircuit targeting could be useful in a clinical population, raising the idea that mPFC might be an important neuromodulation target for people with psychosis spectrum illness, especially in early phases. For instance, [121] applied deep brain stimulation (DBS) to mPFC in adolescent rats from the neurodevelopmental maternal immune stimulation model of schizophrenia. They found that DBS in this region prevented the development of impaired sensorimotor gating and executive function in adulthood, and normalized dopaminergic and serotonergic transmission indicative of widespread restorative neural plasticity beyond that related to social behavior.

At the very least, one would hypothesize that combining social cognition training with *TMS activation of mPFC* could be useful in enhancing social cognition, mood, motivation and adaptive engagement with treatment. *TMS inhibition of mPFC* could be useful for addressing the biasing effect of negative memory schemas, which probably play a role in clinical symptoms of defeatist beliefs and possibly also delusions. In a study of individuals with depression, TMS inhibition of mPFC resulted in a lower false recognition of negative critical lures, suggesting that such an approach is feasible [122].

## Dysregulated experience-dependent plasticity processes interact across levels of scale and time and include compensatory mechanisms that have pathogenic importance

The timescales of the factors which operate on and affect plasticity processes range from milliseconds to hours, days, years, and even intergenerationally. In rat experiments, stressful postnatal experiences, for example, impair memory performance and hippocampal plasticity not just in the animals but *in their future offspring*, a finding with sobering implications for social determinants of psychosis risk [123–125]. We highlight a few of these dynamic factors that are relevant to pathogenesis and intervention development.

### Deleterious experiences such as cannabis exposure or persistent stress can affect the plasticity machinery, which is itself plastic

A common exogenous factor that increases the risk for psychosis and affects the plasticity machinery is cannabis use. Miller et al. have recently shown, in a rat model, that adolescent THC exposure induces premature and irreversible pruning of dendritic spines, protracted atrophy of apical trees, and significant changes in the transcriptional patterns of pyramidal neurons [126]. Specifically, adolescent THC exposure alters the gene networks involved in the regulation of cytoskeletal dynamics at excitatory synapses and dendritic spines (as well as gene networks involved in epigenetic histone and chromatin modifications). Taken together, their results indicate that THC in the adolescent brain prematurely attenuates plasticity mechanisms in circuits that are refining themselves for adulthood, while also showing evidence of long-lasting changes in pyramidal cell function.

Stress, which is also related to the risk for psychosis, alters neuroplasticity operations in multiple complex ways via its effects on noradrenergic and dopaminergic neurotransmission and on the HPA axis (see refs. [28, 127–129]). As noted earlier, people who develop psychosis have a higher rate of exposure to adverse childhood events, which primes them for sensitization and dysregulation of midbrain dopamine systems in response to future acute stressors; the increased stress responsivity interacts with pre-existing neurodevelopmental and cognitive vulnerabilities and peaks in adolescence, facilitating the conditions for the onset of psychosis [51].

Stress and its biological effects can both accelerate or delay critical periods of heightened plasticity depending on the timing and nature of the stressor [130]. In animal models, maternal and perinatal immune challenge [131], parental separation [132]), and social isolation [133] all lead to anomalies in fast-spiking parvalbumin-expressing (PV) interneuron cells in prefrontal cortex and/or in hippocampus. Maturation of these inhibitory PV interneurons—along with the maturation of the perineuronal nets (PNNs) which enwrap them—is required for the closing of critical periods in interaction with microglial—mediated synaptic pruning [22, 134]. All of these processes are modified by experience-induced neural activity and are affected by chronic stress.

#### Changes in PV and PNN maturation

Without the appropriate experiential inputs during critical periods, the number and strength of PV synapses on pyramidal cells remain reduced; PV cell membranes fail to mature; and perineuronal net (PNN) growth is delayed, with the net result being immature cortical circuitry [135]. Chronic stress exposure affects this PV and PNN maturation: a recent study of prolonged stress in adolescent rats showed that PNN maturation was slowed in medial and orbito-frontal cortices and that frontally-mediated behaviors were impaired. Another function of PNNs is to protect PV cells from oxidative stress, but PNNs can themselves be damaged by excessive redox dysregulation, which then results in PNN loss and prolongs critical period plasticity or circuit immaturity [136]; this process has been proposed to contribute to circuit instability in schizophrenia [137].

#### Changes in microglial activity

Microglia respond to changes in neural activity by harnessing inflammatory signaling cascades to assemble, remodel, and eliminate synapses across the entire lifespan [85]. Increased endogenous microglia engulfment of synapses (for example, due to a genetic predisposition to exagerrated complement synapse-tagging [37]) may be one explanation for the decrease in dendritic spine density, the reduced gray matter, and the functional dysconnectivity seen in schizophrenia [138]. However, complex bidirectional interactions between physiological state, neuronal activity, and microglial process dynamics are the rule. Pre- and postnatal early life stress as well as stressors in adulthood can prime microglial reactivity [139], leading to maladaptive synaptic remodeling and/or proinflammatory processes that can last throughout the lifespan [140].

In sum, the brain retains a lifelong capacity to toggle back and forth between a less-plastic or more-plastic state as a function of PV interneuron function, PNN integrity, synaptic remodeling, and microglial activity—processes which are exquisitely sensitive to not just microcircuit E-I balance and neuronal activity levels, but also to early life events, exposures to substances such as cannabis, and the consequences of psychological stress such as altered neurohormonal activity and redox dysregulation [137, 140, 141].

### Chronic unreliable (noisy) inputs dysregulates critical period plasticity mechanisms

Prolonged exposure to unstructured stimuli—unreliable or noisy sensory inputs—can have the same effect as deleterious rearing environments in terms of derailing the trajectory of critical period plasticity. For example, exposing juvenile rat pups to temporally modulated white noise produces a shorter than usual critical period for spectral tuning in the auditory cortex, while masking normal auditory inputs with continuous white noise keeps it open indefinitely [142]. As noted earlier, even in the mature cortex, sensory inputs with low signal-to-noise ratios can maladaptively open plasticity windows, as has been shown in young adult rats housed in a noisy auditory environment for 8 weeks [67]. These young adult animals show neuronal desynchronization, poor tuning selectivity, and reduced sensitivity to low-probability sounds, associated with reduced inhibitory interneuron expression and decreased cortical myelin. Noisy sensory inputs, whether originating from the environment or from endogenous factors associated with aging or with pathology, drive inhibitory interneuron functioning “backwards” and stimulate microglial synaptic engulfment to generate functional and structural changes consistent with immature circuit function.

What are the implications for psychosis spectrum illnesses? If the cortex is exhibiting E-I imbalance, manifesting prediction errors, and functioning as an unreliable or noisy sensory processor, this will result in a subtle degradation over time in the statistical structure and fidelity of sensory inputs and in the predictive machinery that resolves them; indeed, during the height of acute psychosis, such statistical structure and fidelity is entirely disrupted. This may toggle the prefrontal cortex into a state of heightened (dysregulated, immature) plasticity. Abnormally elevated neuromodulatory function may compound this state. Similar to the aging brain, the result is not just cognitive impairment, but can include the development of compensatory neural system reorganization that is not always behaviorally adaptive [143]. In adolescent rats, a mere 25% downregulation in PV levels in frontal cortex was sufficient to significantly reduce GABAergic inhibitory transmission in a manner that persisted into adulthood—permanently disrupting prefrontal E-I balance, afferent processing from the ventral hippocampus, and prefrontally-mediated behaviors [144]. These data suggest that adolescence is characterized by a critical window for prefrontal PV interneuron maturation in order to develop the adaptive “inhibitory endophenotype” needed to sustain adult prefrontal functions. If sufficient PV maturation does not occur during this critical window, then long-lasting deficits in prefrontal microcircuit and macrocircit functioning are the consequence.

### Dysregulated critical period plasticity appears to be a feature of psychosis, especially in the early phases

Consistent with an abnormal state of immature plasticity, in a recent bioinformatics study of microarray datasets of schizophrenia and bipolar disorder, Smith et al. found a distinct cluster of 60% of psychosis spectrum individuals who showed a “hyperplasticity phenotype” in their functional plasticity transcriptomics. (They studied the critical period-related transcriptome for ocular dominance plasticity in primary visual cortex, which presumably reflects aspects of transcriptome activity related also to experience-dependent plasticity processes [145]). The rest of the sample showed either a reduced or normal functional plasticity phenotype.

Individuals with schizophrenia also exhibit transcriptional immaturity of prefrontal cortex-expressed genes, such that the transcriptional patterns more closely resemble those of infants 1–5 years old than healthy adults [146]. The expression of genes that are normally increased during human prefrontal development (maturation markers) is decreased, while genes for immaturity markers are relatively increased, though to a lesser degree. The relationship of these findings to illness duration is not known, nor is their relationship to the accelerated brain aging described further below. Individuals with schizophrenia also manifest reduced perineuronal nets, reduced PV interneuron density, and evidence of increased microglial activity [138, 147, 148].

In animal experiments, dysplasticity processes can affect discrete subregions/circuits across a cortical sector, depending on the nature of the abnormal sensory inputs [149]. The same may well be true for prefrontal cortex in humans. Such a mechanism might help to explain why different patterns of symptoms—sensory hallucinations, delusions of control, paranoid beliefs—emerge for different individuals, which could be a function of the underlying prefrontal cortical circuit that is undergoing dysplastic/hyperplastc changes and/or the functional hierarchical differences among cortical sectors [54]. This is consistent with Sterzer et al.’s discussion on how different kinds of prefrontal predictive coding impairments might arise for different sensory modalities and their integration [46].

It’s worth noting that dysregulation of critical period plasticity machinery is also likely in autism spectrum disorders (ASD), but earlier in life [150]. Mouse models of ASD strongly implicate a disruption of plasticity mechanisms that operate during the early sensory critical periods (e.g., [151]), while [152] studies of familial ASD risks converge on structural and functional deficits in visual cortical areas at 6–12 months—a peak of visual cortical plasticity and well before social dysfunction manifests. ASD may be characterized by aberrant plasticity processes manifesting during early critical periods in primary sensory cortices, with a downstream cascading dysregulation in social information processing as the child develops. In contrast, psychosis spectrum illnesses may be more preferentially associated with dysregulation of plasticity mechanisms in prefrontal cortex during the later adolescent critical period of social development—perhaps after a slow cascading cumulation of subtle microcircuit changes. However, the shared genetic risk observed between ASD and psychosis, along with the fact that ASD-like features are seen in approximately 20% of early psychosis individuals, implies an overlap in pathophysiology for a subset of individuals [13].

### Initially dysregulated and immature “hyperplasticity” may be followed by hypoplasticity

Smith et al. [145] also observed that individuals with a shorter duration of illness showed the hyper- critical period transcriptional phenotype; individuals with an intermediate duration of illness showed no change in critical period gene expression; and individuals with a long duration of illness had a hypo- critical period transcriptional phenotype. This suggests that illness duration has a dose-dependent influence on the expression of critical period genes and that earlier phases of illness are characterized by immature or dysregulated “hyperplastic” processes characteristic of an immature prefrontal cortex. At the same time, brain structural changes observed in people with psychosis suggest an accelerated developmental period (see ref. [153]); individuals with schizophrenia reach their point of greatest white matter functional anisotropy six years earlier than controls [154].

**Fig. 3:**
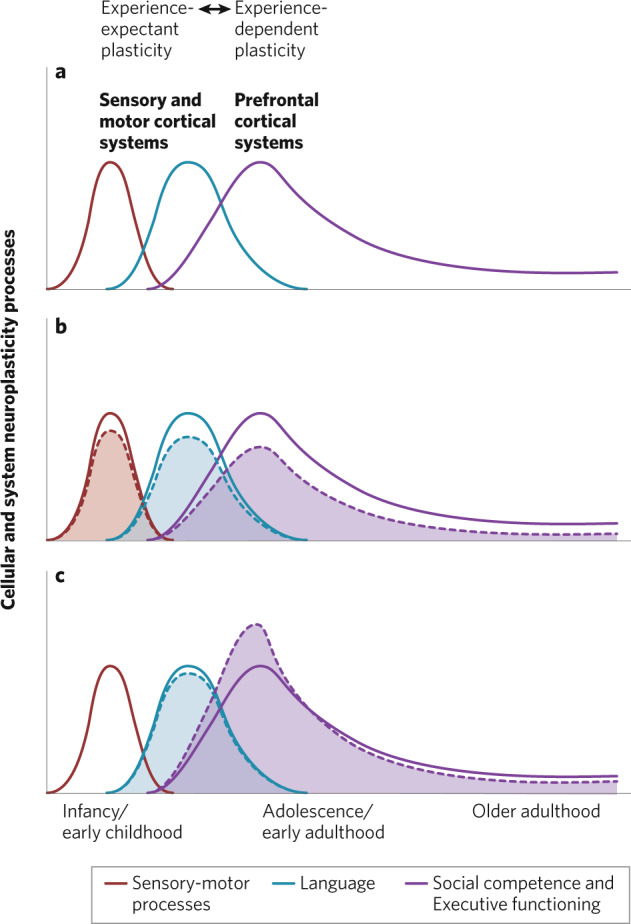
Critical periods of plasticity—from infancy to adulthood—in sensory-motor systems, language, and social competence/ executive functioning. **a** Normal development. **b** Hypothesized *hypoplasticity* psychosis spectrum illness course—Low-level microcircuit anomalies and/or hypoplastic processes are present at birth, subtly affect sensory-motor and language development, accumulate over time, and worsen in adolescence and through adulthood. **c** Hypothesized *hyperplasticity* illness course—Subtle microcircuit dysfunction or hypoplasticity may be present prodromally (e.g., possibly manifesting during language development) but then cascades into cortical hyperexcitability and the sudden dysregulation/upregulation of cortical plasticity mechanisms during adolescence. Secondary hypoplasticity processes ensue. (Adapted with permission from ref. [142]).

A large body of work also shows a consistent pattern of differences in cortical connectivity and activity/excitability between very early and chronic phases of illness, moving from abnormally high to abnormally low. In early illness, patterns of brain-wide resting state functional hyperactivity and hyperconnectivity are observed, especially in the prefrontal cortex (e.g., [155]^,^ and are associated with the severity of positive symptoms [156]. Later in the illness, functional hypoconnectivity is seen (resting state functional connectivity is felt to reflect Hebbian neural system plasticity (Guerra-Carrillo et al., 2014).). The degree of cortical functional hyperactivity in the first episode correlates with the decline in functional connectivity over time (as reviewed in ref. [4]). Dysregulated high frequency oscillatory activity is seen in early phases of the illness [157, 158]; during the clinical high risk period, elevated high-gamma activity, consistent with increased glutamatergic excitability, is observed in frontal and temporal areas, but in later phases of illness it is abnormally reduced [57]. These findings, though not definitive, suggest that a period of dysregulated cortical “hyperplasticity” may be followed later by hypoplasticity in at least some individuals, and may reflect a distinct process from a more persistently “hypoplastic” phenotype (Fig. 3).

What is not at all clear is whether this developmental picture of dysregulated hyperplasticity in adolescence followed by later hypoplasticity is the general rule, or if it reflects just a small piece of a complex picture of genetically-determined hyper/hypo plasticity imbalance that exists in different subgroups of people with psychosis. Certainly, the psychosis biotypes identified by the BSNIP consortium—and the fact that the biotypes hold true in first-degree relatives—suggest this latter model. Biotype 1 individuals manifest cortical hypoexcitability along with significant and widespread reductions in gray matter volume and poorer cognition and functioning [159], consistent with a persistent hypoplasticity syndrome, accelerated aging, and a more relentlessly impairing form of illness [2]. But Biotype 2 individuals show high cortical excitability and less extensive reductions in gray matter volume, suggesting causal roles for psychological stress and trauma, which are associated with intrinsic sensory hyperactivity and bottom-up inhibition deficits [160]. Biotype 2 is also reminiscent of the sensory hyperexcitability and prefrontal hyperconnectivity reported in early phases of psychosis [155, 157], and could be consistent with potential hyperplasticity processes being at play [145].

### Dysregulated plasticity is associated with progressive gray matter loss

If dysregulated or “hyperplastic” critical period processes are occurring in early phases of illness, they are happening concurrently with dynamic macro-scale structural brain changes that signal a devolving cortex, consistent with over-exuberant synaptic sculpting as the brain attempts to shape its representations to an environment with unreliable statistics and/or is responding to increased stress and inflammation. High-risk individuals who develop psychosis have greater rates of cortical atrophy and reductions in the fractional anisotropy of white matter than those who don’t over the one-year period prior to onset of illness [161, 162]. In animal models, sensory deprivation, sensory noise, deleterious environments, and social isolation all impact cortical integrity and myelination [163]. Of note, in high risk individuals who do not develop psychosis, rates of cortical atrophy are also accelerated but to a lesser extent, suggesting the importance of protective or resilience factors in mitigating the transition to psychosis in an otherwise at-risk brain that manifests dysplastic structural changes [161].

After the onset of psychosis, accelerated brain aging continues to be observed, particularly in the first few years. Changes include reduced white and gray matter volume [164] and changes in gray matter density maps [165]. The rate of gray matter volume loss is greatest in young adulthood, consistent with an accelerated synaptic reduction during early illness [166]. In contrast, reductions in white matter functional anisotropy and the rate at which those reductions grow are increased over the lifespan, consistent with a brain that is gradually becoming “unwired” (losing integrity in long range communication pathways) in response to altered cortical processing [154, 166]. Similar findings are seen across the population as a function of genetic risk, suggesting again that adolescence critical period dysplasticity mechanisms are a permissive rather than causal factor and that resilience or protective factors are important [167].

Increases in inflammatory signaling, which as mentioned earlier are known to impact synaptic pruning [168], may play a role in this accelerated aging process. Proinflammatory plasma cytokines predict the future one-year gray matter loss in high-risk individuals, as do a briefer duration of prodromal symptoms [161]. This clinical picture of *acute symptom onset* + *elevated inflammatory processes* + *accelerated gray matter loss* may represent a group of individuals who have rapidly entered a period of hyperexcitability-induced dysregulated plasticity and cortical failure, as compared to individuals with an insidious symptom onset with less acutely accelerated gray matter loss, who may be characterized by long-standing hypoplastic processes dating back to childhood or earlier (Fig. 3). Inflammatory dysregulation could be an important causal factor for the first group, in which case it might drive dysplasticity via its influence on oxidative stress, PNNs, and microglial functioning, or could be secondary to altered cortical efficiency (acute onset of significant excitability/neuronal ensemble decorrelation) and the experience of an unpredictable and persistently stressful environment.

Generally speaking, however, by the time the illness has entered chronic phases, the picture is one of gray matter loss and widespread impaired plasticity responses within and across neural circuits [169]. Degeneration of neuromodulatory function is likely a major contributor to the diminished plasticity. While we discussed dopamine’s pro-plasticity effects in Section “Experience-dependent dysplasticity in psychosis emerges from activity decorrelation within neuronal ensembles”, cholinergic and noradrenergic neuromodulation are also key regulators of plasticity, facilitating cue detection, attentional control, molecular synaptic plasticity, and thalamo-cortical connectivity [170, 171]. Cholinergic and noradrenergic tone are reduced in the illness and all three neuromodulatory systems are affected by the medications that are commonly prescribed. Anticholinergic medication burden in particular has a strong deleterious affect on cognition and cortical function and is associated with a reduced response to neuroplasticity-informed cognitive training [172, 173].

### Compensatory plasticity processes are the rule

In the face of both pathologic challenges and normal experience-dependent changes, the brain engages in functional adaptations to maintain the stability and functional capacity of neural networks. Compensatory plasticity mechanisms can occur across different levels of scale, with different temporal trajectories and downstream effects, and with different pathogenic implications. We highlight several examples.

#### Compensation at the microcircuit level

[152] performed dynamic causal modeling on EEG (resting state, ASSR, MMN) and on resting state fMRI data from individuals with schizophrenia and found that the experimental data were best accounted for by a single underlying parameter change: greater self-inhibition in excitatory pyramidal cells. However, they also found that this primary loss of synaptic gain on pyramidal cells appeared to be compensated for by a secondary downregulation of inhibitory interneurons, and that psychotic symptoms were the result of this secondary downregulation.

#### Compensation at the systems level

[4] have posited that “compensatory” homeostatic plasticity processes explain the progressive loss of neural network structure and function that occurs over time in schizophrenia. Consistent with [174], they suggest that initial microcircuit hyperactivation triggers functional and possibly structural synaptic downregulation that then cascades from the microcircuit to the macrocircuit level, resulting in widespread prefrontal functional disconnectivity as a homeostatic process. During a spatial memory task, as an example, theta phase coupling between mPFC and medial temporal lobe is impaired in people with schizophrenia and does not show the normal association with task performance that it does in controls. The medial temporal regions implicated overlap with regions showing reduced GABA_A_ receptor availablility, suggesting a role for reduced inhibitory tone in this process [175].

Prefrontal disconnectivity, including prefrontal-thalamic disconnectivity, coexists with *hyper*connectivity between thalamus and sensory cortices [176, 177]. This signature is increased in individuals who are at clinical high-risk for psychosis, with the most pronounced changes being in individuals who will later develop illness [178]. Prefrontal-thalamic circuits are uniquely equipped to support cognitive functions which require extended cortical representations over time. A recent study [179] employed chemogenetic circuit suppression in maturing mice to demonstrate that activation of thalamo-prefrontal circuits during the adolescent critical period but not in adulthood was necessary to establish normal cognitive function and behavior. If these circuits are functionally impaired, the brain may attempt to co-opt plasticity in thalamo-sensory networks in an attempt to compensate for prefrontal dysfunction, but such compensation is unlikely to be completely successful. Schmitt et al have shown in rodents that mediodorsal thalamo-prefrontal inputs can amplify local prefrontal connectivity in a supralinear way, but lateral geniculate thalamo-visual cortical inputs only amplify in a sublinear fashion [180].

Consistent with these ideas, in an MEG study of the encoding of speech sounds in people with schizophrenia, participants with schizophrenia did not show the normal correlation of task performance with task-induced high-gamma power in superior temporal gyrus that was observed in healthy subjects [181]. Instead, they recruited the left visual word form area (VWFA). VWFA activity during encoding correlated with a neuropsychological measure of auditory working memory and also with the magnitude of hallucinations. It is plausible that left VWFA plasticity was harnessed to compensate for dysfunction in superior temporal gyrus in people with high hallucinations, suggesting a partially successful functional reorganization in visual association cortex in response to abnormal neuronal dynamics in the temporal sector.

A deeper understanding of compensatory network changes is likely to be important for optimal treatment development. [182] has shown abnormally increased thalamo-temporal connectivity at baseline in young recent-onset individuals, consistent with the literature, but also found that after intensive cognitive training of the auditory system, this connectivity was *increased even further* compared to the control group. Moreover, the increase in thalamo-temporal connectivity was positively associated with post-training gains in cognition. This suggests that training (or other corrective experiences) may sometimes harness a compensatory pathway to drive behavioral improvements, while in other instances, compensations may result in further functional or behavioral impairment.

### Plasticity-informed treatment approaches to address unmet treatment needs

The most important principle to emerge from this developmental theme is “The right intervention for the right process at the right time.” A second principle, which so far has not received much focused research attention, is the clear role for protective or resilience factors: if progressive gray matter loss and abnormally increased thalamo-cortical connectivity are observed in clinical high risk individuals and in individuals with high genetic loading *who do not develop psychosis spectrum illness*, then what is protecting them? Can we deliberately harness these protective factors? Finally, the noisy/unreliable cortical dynamics and hypoplasticity seen in persistent psychosis, along with indices of accelerated brain aging, suggest that we may gain useful insights from considering senescence mechanisms in the brain as another window into the illness trajectory and avenue for intervention. For all of these questions, we need a better understanding of the reverberating interactions linking primary and downstream pathogenic processes to each other, to the brain’s plasticity responses, and to clinical symptoms, before we can create a precise map for the nature and timing of optimal interventions. But a few ideas are evident and either have an emerging evidence-base or are ripe for further investigation (Table 3).

**Table 3:** A neuroplasticity-based perspective on near-term clinical practice and clinical research directions for psychosis spectrum illnesses.

Mechanism of action	Phase of Illness/Illness biotype to be targeted	Potential treatment approaches
Reduce antiplasticity factors	Likely relevant to all individuals experiencing psychosis spectrum illness May need special considerations for the subgroup of individuals characterized by neuroinflammation?	Eliminate or reduce medication-induced anticholinergic burden through all possible prescribing approaches Intervene to avoid or mitigate social deafferentiation and maintain appropriate levels of social/cognitive stimulation *Vigorously address obesity and poor dietary practices/dysbiosis/systemic inflammation* [183] *Since both immune activation and suppression impair plasticity* [197], *need judicious use of anti-inflammatory agents, especially in early phases* [198]
Increase pro-plasticity factors	Likely most relevant for individuals who are in ongoing phases of illness and/or for biotypes with longstanding premorbid cognitive impairment, prefrontal dysfunction, cortical hypoexcitability, and hypotheized hypoplasticity	Offer cognitive and social cognitive training, psycho-social therapies Use neuromodulation and/or pharmacotherapy to enhance cognitive training effects Encourage regular aerobic exercise *Consider prescribing pro-plasticity agents: e.g. metformin, valproic acid or other HDAC inhibitors, D-serine, cholinergic M4 allosteric modulators* [198–201] *Early music training may serve as a long-term protective intervention* [186]
Modulate the trajectory of prefrontal critical period plasticity/dysregulated plasticity	Likely most relevant for a subgroup of individuals who are late in the clinical high-risk period or who are in their first episode of psychosis without evidence of long premorbid decline May include biotypes without marked premorbid cognitive impairment who show cortical hyperexcitability and hypothesized hyperplasticity Might be important in early-phase individuals who have marked neuroinflammation?	Judicious use of GABA-ergic medications that reduce cortical excitability and reverse dysregulated plasticity responses (e.g., benzodiazepines, possibly gabapentin) *Prescribe antioxidants to mitigate dysplasticity effects of oxidative stress* [202] *Develop an in-depth understanding of psychosocial and biological protective factors in clinical high-risk individuals who manifest brain changes but do not transition into psychosis*

Italics indicate plausible approaches that have an emerging evidence-base but require additional research. The optimal timing and personalization of these approaches will require a deeper knowlege of the abnormalities in plasticity mechanisms that occur within and across subgroups of individuals and also as someone moves from the clinical high risk phase into a defined episode of psychosis.

For example, all individuals with psychosis spectrum illness are likely to benefit from approaches which reduce anti-cognitive and anti-plasticity factors. Foremost among these is changing our prescribing practices to reduce (whenever possible) anticholinergic burden. Other interventions include a therapeutic focus on adaptive social and cognitive stimulation and a vigorous approach to reducing obesity and improving nutrition; findings are coming to light on the associations among the traditional Western diet, dysbiosis, inflammation, and reduced neuroplasticity [183].

In people with more persistent illness showing features of ongoing hypoplasticity, a wide range of pro-plasticity interventions will be important—to include not just cognitive and social-cognitive training and psychosocial therapies, but also physical exercise, which has a strong evidence-base but is not yet a primary focus of most treatment programs [184]. Additional research will determine how to optimally apply these interventions in conjunction with pro-plasticity medications and neuromodulation. The anti-glycemic anti-senescence medication metformin induces expression of BDNF and reduces stress-induced behavior in mice, suggesting its potential usefulness as an adaptive pro-plasticity agent [185]. Valproic acid and other HDAC-inhibitors, D-serine, and M4 allosteric modulators all have pro-plasticity effects as well, as does early-life music training [186].

There appears to be a subgroup of individuals with a more acute onset and evidence of abruptly dysregulated plasticity, possibly including significant inflammatory signaling, oxidative stress, and consequent dysfunction in PV cells, devolving PNN integrity, and increased synaptic pruning that leads to progressively worse downstream cognitive effects [187]. For these individuals, modulating those processes as soon as possible will be important [136]. This will include the vigorous early use of antioxidants and molecular brakes that can close runaway or dysregulated critical period plasticity [188], such as the judicious use of medications that enhance inhibitory GABA-ergic tone. Specific intensive cognitive training and social skills-focused psychosocial treatment are also known to protect against the progressive gray matter loss seen in early illness, as unequivocally shown in the work of [72, 109, 189], and can be enhanced by pro-cognitive medications as well as neuromodulation. The search for other protective factors—endogenous and exogenous—should be a major focus of investigation.

## Future research directions

A number of near-term and long-term research questions and treatment implications emerge from the neuroplasticity lens we have used in this review. They range from actions which clinicians can implement immediately, such as reducing the use of anticholinergic medications as much as possible [173] and thoughtfully considering the off-label use of anti-inflammatory and anti-oxidant agents in early illness [190], to long-term gene-expression modification therapies.

High-yield insights will come from basic science experiments that delineate: (1) How both exogenous and endogenous factors contribute to the dysregulation of prefrontal plasticity during adolescence; and, (2) How this process drives micro-circuit and macro-circuit changes related to hyperexcitability, impaired interneuron inhibition and PNN maturation, microglial activity, and subsequent hyperplastic/dysplastic changes in brain structure and connectivity. Computational models will need to account for the reverberating nature of aberrant plasticity processes that occur at all levels of the brain’s information processing hierarchy. Experimental work will need to probe the unique nature of plasticity changes that may occur in functionally distinct prefrontal circuits and the compensatory mechanisms that arise as a consequence. Well-designed basic science experiments that study perturbations in plasticity mechanisms at various levels of scale, supported by algorithmic and neurophysiological computational modeling that are applied to longitudinal human data, will help to shed light on these complex interacting processes.

## References

[1] Meyer-Lindenberg A, Tost H (2014). Neuroimaging and plasticity in schizophrenia. Restor Neurol Neurosci.

[2] Forsyth JK, Lewis DA (2017). Mapping the Consequences of Impaired Synaptic Plasticity in Schizophrenia through Development: An Integrative Model for Diverse Clinical Features. Trends Cogn Sci.

[3] Keshavan MS, Mehta UM, Padmanabhan JL, Shah JL (2015). Dysplasticity, metaplasticity, and schizophrenia: Implications for risk, illness, and novel interventions. Dev Psychopathol.

[4] Krystal JH, Anticevic A, Yang GJ, Dragoi G, Driesen NR, Wang X-J (2017). Impaired Tuning of Neural Ensembles and the Pathophysiology of Schizophrenia: A Translational and Computational Neuroscience Perspective. Biol Psychiatry.

[5] Stephan KE, Friston KJ, Frith CD (2009). Dysconnection in schizophrenia: from abnormal synaptic plasticity to failures of self-monitoring. Schizophr Bull.

[6] Balu DT, Coyle JT (2011). Neuroplasticity signaling pathways linked to the pathophysiology of schizophrenia. Neurosci Biobehav Rev.

[7] Hamm JP, Peterka DS, Gogos JA, Yuste R (2017). Altered Cortical Ensembles in Mouse Models of Schizophrenia. Neuron.

[8] Zick JL, Crowe DA, Blackman RK, Schultz K, Bergstrand DW, DeNicola AL (2022). Disparate insults relevant to schizophrenia converge on impaired spike synchrony and weaker synaptic interactions in prefrontal local circuits. Curr Biol.

[9] Lee H, Dvorak D, Fenton AA (2014). Targeting Neural Synchrony Deficits is Sufficient to Improve Cognition in a Schizophrenia-Related Neurodevelopmental Model. Front Psychiatry.

[10] O’Donnell C, Gonçalves JT, Portera-Cailliau C, Sejnowski TJ. Beyond excitation/inhibition imbalance in multidimensional models of neural circuit changes in brain disorders. Elife. 2017;6. 10.7554/eLife.26724. 10.7554/eLife.26724 PMC5663477 29019321

[11] Hensch TK, Fagiolini M (2005). Excitatory-inhibitory balance and critical period plasticity in developing visual cortex. Prog Brain Res.

[12] Fagiolini M, Katagiri H, Miyamoto H, Mori H, Grant SGN, Mishina M (2003). Separable features of visual cortical plasticity revealed by N-methyl-D-aspartate receptor 2A signaling. Proc Natl Acad Sci USA.

[13] Singh T, Poterba T, Curtis D, Akil H, Al Eissa M, Barchas JD (2022). Rare coding variants in ten genes confer substantial risk for schizophrenia. Nature.

[14] Trubetskoy V, Pardiñas AF, Qi T, Panagiotaropoulou G, Awasthi S, Bigdeli TB (2022). Mapping genomic loci implicates genes and synaptic biology in schizophrenia. Nature.

[15] Cisneros-Franco JM, Ouellet L, Kamal B, de Villers-Sidani E. A Brain without Brakes: Reduced Inhibition Is Associated with Enhanced but Dysregulated Plasticity in the Aged Rat Auditory Cortex. eNeuro. 2018;5. 10.1523/ENEURO.0051-18.2018. 10.1523/ENEURO.0051-18.2018 PMC6140119 30225357

[16] Dan Y, Poo M-M (2004). Spike timing-dependent plasticity of neural circuits. Neuron.

[17] Zick JL, Blackman RK, Crowe DA, Amirikian B, DeNicola AL, Netoff TI (2018). Blocking NMDAR Disrupts Spike Timing and Decouples Monkey Prefrontal Circuits: Implications for Activity-Dependent Disconnection in Schizophrenia. Neuron.

[18] Selemon LD (2013). A role for synaptic plasticity in the adolescent development of executive function. Transl Psychiatry.

[19] Sheridan SD, Horng JE, Perlis RH. Patient-Derived In Vitro Models of Microglial Function and Synaptic Engulfment in Schizophrenia. Biol Psychiatry. 2022. 10.1016/j.biopsych.2022.01.004. 10.1016/j.biopsych.2022.01.004 PMC10039432 35232567

[20] Hanson KL, Grant SE, Funk LH, Schumann CM, Bauman MD. Impact of Maternal Immune Activation on Nonhuman Primate Prefrontal Cortex Development: Insights for Schizophrenia. Biol Psychiatry. 2022. 10.1016/j.biopsych.2022.04.004. 10.1016/j.biopsych.2022.04.004 PMC9888668 35773097

[21] Fernandez A, Meechan DW, Karpinski BA, Paronett EM, Bryan CA, Rutz HL (2019). Mitochondrial Dysfunction Leads to Cortical Under-Connectivity and Cognitive Impairment. Neuron.

[22] Schafer DP, Lehrman EK, Kautzman AG, Koyama R, Mardinly AR, Yamasaki R (2012). Microglia sculpt postnatal neural circuits in an activity and complement-dependent manner. Neuron.

[23] Dienel SJ, Schoonover KE, Lewis DA. Cognitive Dysfunction and Prefrontal Cortical Circuit Alterations in Schizophrenia: Developmental Trajectories. Biol Psychiatry. 2022. 10.1016/j.biopsych.2022.03.002. 10.1016/j.biopsych.2022.03.002 PMC9420748 35568522

[24] Yagishita S, Hayashi-Takagi A, Ellis-Davies GCR, Urakubo H, Ishii S, Kasai H (2014). A critical time window for dopamine actions on the structural plasticity of dendritic spines. Science.

[25] He K, Huertas M, Hong SZ, Tie X, Hell JW, Shouval H (2015). Distinct Eligibility Traces for LTP and LTD in Cortical Synapses. Neuron.

[26] Kasai H, Ziv NE, Okazaki H, Yagishita S, Toyoizumi T (2021). Spine dynamics in the brain, mental disorders and artificial neural networks. Nat Rev Neurosci.

[27] Howes OD, Hird EJ, Adams RA, Corlett PR, McGuire P (2020). Aberrant Salience, Information Processing, and Dopaminergic Signaling in People at Clinical High Risk for Psychosis. Biol Psychiatry.

[28] Howes OD, McCutcheon R, Owen MJ, Murray RM (2017). The Role of Genes, Stress, and Dopamine in the Development of Schizophrenia. Biol Psychiatry.

[29] Tseng H-H, Watts JJ, Kiang M, Suridjan I, Wilson AA, Houle S (2018). Nigral Stress-Induced Dopamine Release in Clinical High Risk and Antipsychotic-Naïve Schizophrenia. Schizophr Bull.

[30] Abi-Dargham A, van de Giessen E, Slifstein M, Kegeles LS, Laruelle M (2009). Baseline and Amphetamine-Stimulated Dopamine Activity Are Related in Drug-Naïve Schizophrenic Subjects. Biol Psychiatry.

[31] Urakubo H, Yagishita S, Kasai H, Ishii S (2020). Signaling models for dopamine-dependent temporal contiguity in striatal synaptic plasticity. PLoS Comput Biol.

[32] Slifstein M, van de Giessen E, Van Snellenberg J, Thompson JL, Narendran R, Gil R (2015). Deficits in prefrontal cortical and extrastriatal dopamine release in schizophrenia: a positron emission tomographic functional magnetic resonance imaging study. JAMA Psychiatry.

[33] Rao N, Northoff G, Tagore A, Rusjan P, Kenk M, Wilson A (2019). Impaired Prefrontal Cortical Dopamine Release in Schizophrenia During a Cognitive Task: A [11C]FLB 457 Positron Emission Tomography Study. Schizophr Bull.

[34] Frankle WG, Himes M, Mason NS, Mathis CA, Narendran R. Prefrontal and Striatal Dopamine Release Are Inversely Correlated in Schizophrenia. Biol Psychiatry. 2022. 10.1016/j.biopsych.2022.05.009. 10.1016/j.biopsych.2022.05.009 35791965

[35] Timms AE, Dorschner MO, Wechsler J, Choi KY, Kirkwood R, Girirajan S (2013). Support for the N-methyl-D-aspartate receptor hypofunction hypothesis of schizophrenia from exome sequencing in multiplex families. JAMA Psychiatry.

[36] Kirov G, Pocklington AJ, Holmans P, Ivanov D, Ikeda M, Ruderfer D (2012). De novo CNV analysis implicates specific abnormalities of postsynaptic signalling complexes in the pathogenesis of schizophrenia. Mol Psychiatry.

[37] Sekar A, Bialas AR, de Rivera H, Davis A, Hammond TR, Kamitaki N (2016). Schizophrenia risk from complex variation of complement component 4. Nature.

[38] Glantz LA, Lewis DA (2000). Decreased dendritic spine density on prefrontal cortical pyramidal neurons in schizophrenia. Arch Gen Psychiatry.

[39] MacDonald ML, Alhassan J, Newman JT, Richard M, Gu H, Kelly RM (2017). Selective Loss of Smaller Spines in Schizophrenia. Am J Psychiatry.

[40] Jauhar S, Nour MM, Veronese M, Rogdaki M, Bonoldi I, Azis M (2017). A Test of the Transdiagnostic Dopamine Hypothesis of Psychosis Using Positron Emission Tomographic Imaging in Bipolar Affective Disorder and Schizophrenia. JAMA Psychiatry.

[41] Kegeles LS, Abi-Dargham A, Frankle WG, Gil R, Cooper TB, Slifstein M (2010). Increased synaptic dopamine function in associative regions of the striatum in schizophrenia. Arch Gen Psychiatry.

[42] Howes OD, Bose SK, Turkheimer F, Valli I, Egerton A, Valmaggia LR (2011). Dopamine Synthesis Capacity Before Onset of Psychosis: A Prospective [18F]-DOPA PET Imaging Study. AJP.

[43] Tang W, Choi EY, Heilbronner SR, Haber SN (2021). Nonhuman primate meso-circuitry data: a translational tool to understand brain networks across species. Brain Struct Funct.

[44] Haber SN, Calzavara R (2009). The cortico-basal ganglia integrative network: the role of the thalamus. Brain Res Bull.

[45] Murray JD, Anticevic A, Gancsos M, Ichinose M, Corlett PR, Krystal JH (2014). Linking microcircuit dysfunction to cognitive impairment: effects of disinhibition associated with schizophrenia in a cortical working memory model. Cereb Cortex.

[46] Sterzer P, Adams RA, Fletcher P, Frith C, Lawrie SM, Muckli L, et al. The Predictive Coding Account of Psychosis. Biol Psychiatry. 2018. 10.1016/j.biopsych.2018.05.015. 10.1016/j.biopsych.2018.05.015 PMC6169400 30007575

[47] Goldman-Rakic PS (1994). Working memory dysfunction in schizophrenia. J Neuropsychiatry Clin Neurosci.

[48] McCutcheon RA, Abi-Dargham A, Howes OD (2019). Schizophrenia, Dopamine and the Striatum: From Biology to Symptoms. Trends Neurosci.

[49] Vinogradov S, Hamid A, Redish D. Etiopathogenic models of psychosis spectrum illnesses must resolve four key questions. Biol Psych. 2022;92:514–22. 10.1016/j.biopsych.2022.06.024 PMC9809152 35931575

[50] Kim IH, Rossi MA, Aryal DK, Racz B, Kim N, Uezu A (2015). Spine pruning drives antipsychotic-sensitive locomotion via circuit control of striatal dopamine. Nat Neurosci.

[51] Varese F, Smeets F, Drukker M, Lieverse R, Lataster T, Viechtbauer W (2012). Childhood adversities increase the risk of psychosis: a meta-analysis of patient-control, prospective- and cross-sectional cohort studies. Schizophr Bull.

[52] Fenton AA (2015). Excitation-inhibition discoordination in rodent models of mental disorders. Biol Psychiatry.

[53] Azouz R (2005). Dynamic spatiotemporal synaptic integration in cortical neurons: neuronal gain, revisited. J Neurophysiol.

[54] Yang GJ, Murray JD, Wang X-J, Glahn DC, Pearlson GD, Repovs G (2016). Functional hierarchy underlies preferential connectivity disturbances in schizophrenia. Proc Natl Acad Sci USA.

[55] Durstewitz D, Seamans JK (2008). The dual-state theory of prefrontal cortex dopamine function with relevance to catechol-o-methyltransferase genotypes and schizophrenia. Biol Psychiatry.

[56] Adams RA, Stephan KE, Brown HR, Frith CD, Friston KJ (2013). The computational anatomy of psychosis. Front Psychiatry.

[57] Grent-’t-Jong T, Gajwani R, Gross J, Gumley AI, Krishnadas R, Lawrie SM (2021). 40-Hz Auditory Steady-State Responses Characterize Circuit Dysfunctions and Predict Clinical Outcomes in Clinical High-Risk for Psychosis Participants: A Magnetoencephalography Study. Biol Psychiatry.

[58] O’Donnell BF, Vohs JL, Krishnan GP, Rass O, Hetrick WP, Morzorati SL (2013). The auditory steady-state response (ASSR): a translational biomarker for schizophrenia. Suppl Clin Neurophysiol.

[59] Thuné H, Recasens M, Uhlhaas PJ (2016). The 40-Hz Auditory Steady-State Response in Patients With Schizophrenia: A Meta-analysis. JAMA Psychiatry.

[60] Koshiyama D, Thomas ML, Miyakoshi M, Joshi YB, Molina JL, Tanaka-Koshiyama K (2021). Hierarchical Pathways from Sensory Processing to Cognitive, Clinical, and Functional Impairments in Schizophrenia. Schizophr Bull.

[61] Thomas ML, Green MF, Hellemann G, Sugar CA, Tarasenko M, Calkins ME (2017). Modeling Deficits From Early Auditory Information Processing to Psychosocial Functioning in Schizophrenia. JAMA Psychiatry.

[62] Javitt DC (2015). Neurophysiological models for new treatment development in schizophrenia: early sensory approaches. Ann N. Y Acad Sci.

[63] Greenwood L-M, Leung S, Michie PT, Green A, Nathan PJ, Fitzgerald P (2018). The effects of glycine on auditory mismatch negativity in schizophrenia. Schizophr Res.

[64] Kantrowitz JT, Epstein ML, Lee M, Lehrfeld N, Nolan KA, Shope C (2018). Improvement in mismatch negativity generation during d-serine treatment in schizophrenia: Correlation with symptoms. Schizophr Res.

[65] Kantrowitz JT, Swerdlow NR, Dunn W, Vinogradov S (2018). Auditory System Target Engagement During Plasticity-Based Interventions in Schizophrenia: A Focus on Modulation of N-Methyl-D-Aspartate-Type Glutamate Receptor Function. Biol Psychiatry Cogn Neurosci Neuroimaging.

[66] Dallérac G, Li X, Lecouflet P, Morisot N, Sacchi S, Asselot R, et al. Dopaminergic neuromodulation of prefrontal cortex activity requires the NMDA receptor coagonist d-serine. Proc Natl Acad Sci USA. 2021;118. 10.1073/pnas.2023750118. 10.1073/pnas.2023750118 PMC8201892 34083436

[67] Kamal B, Holman C, de Villers-Sidani E (2013). Shaping the aging brain: role of auditory input patterns in the emergence of auditory cortical impairments. Front Syst Neurosci.

[68] de Villers-Sidani E, Alzghoul L, Zhou X, Simpson KL, Lin RCS, Merzenich MM (2010). Recovery of functional and structural age-related changes in the rat primary auditory cortex with operant training. Proc Natl Acad Sci USA.

[69] Merzenich MM, Van Vleet TM, Nahum M (2014). Brain plasticity-based therapeutics. Front Hum Neurosci.

[70] Molina JL, Thomas ML, Joshi YB, Hochberger WC, Koshiyama D, Nungaray JA (2020). Gamma oscillations predict pro-cognitive and clinical response to auditory-based cognitive training in schizophrenia. Transl Psychiatry.

[71] Biagianti B, Roach BJ, Fisher M, Loewy R, Ford JM, Vinogradov S, et al. Trait aspects of auditory mismatch negativity predict response to auditory training in individuals with early illness schizophrenia. Neuropsychiatr Electrophysiol. 2017;3. 10.1186/s40810-017-0024-9. 10.1186/s40810-017-0024-9 PMC5568850 28845238

[72] Ramsay IS, Fryer S, Boos A, Roach BJ, Fisher M, Loewy R (2018). Response to Targeted Cognitive Training Correlates with Change in Thalamic Volume in a Randomized Trial for Early Schizophrenia. Neuropsychopharmacology.

[73] Subramaniam K, Luks TL, Garrett C, Chung C, Fisher M, Nagarajan S (2014). Intensive cognitive training in schizophrenia enhances working memory and associated prefrontal cortical efficiency in a manner that drives long-term functional gains. Neuroimage.

[74] Subramaniam K, Luks TL, Fisher M, Simpson GV, Nagarajan S, Vinogradov S (2012). Computerized cognitive training restores neural activity within the reality monitoring network in schizophrenia. Neuron.

[75] Roach BJ, Ford JM, Biagianti B, Hamilton HK, Ramsay IS, Fisher M (2019). Efference copy/corollary discharge function and targeted cognitive training in patients with schizophrenia. Int J Psychophysiol.

[76] Dale CL, Brown EG, Fisher M, Herman AB, Dowling AF, Hinkley LB (2016). Auditory Cortical Plasticity Drives Training-Induced Cognitive Changes in Schizophrenia. Schizophr Bull.

[77] Loewy R, Fisher M, Ma S, Carter C, Ragland JD, Niendam TA (2022). Durable Cognitive Gains and Symptom Improvement Are Observed in Individuals With Recent-Onset Schizophrenia 6 Months After a Randomized Trial of Auditory Training Completed Remotely. Schizophr Bull.

[78] Panizzutti R, Fisher M, Garrett C, Man WH, Sena W, Madeira C (2019). Association between increased serum d-serine and cognitive gains induced by intensive cognitive training in schizophrenia. Schizophr Res.

[79] Vinogradov S, Fisher M, Holland C, Shelly W, Wolkowitz O, Mellon SH (2009). Is serum brain-derived neurotrophic factor a biomarker for cognitive enhancement in schizophrenia?. Biol Psychiatry.

[80] Chow BY, Boyden ES (2013). Optogenetics and translational medicine. Sci Transl Med.

[81] Carrillo-Reid L, Yang W, Bando Y, Peterka DS, Yuste R (2016). Imprinting and recalling cortical ensembles. Science.

[82] Burrows EL, Hannan AJ (2016). Cognitive endophenotypes, gene-environment interactions and experience-dependent plasticity in animal models of schizophrenia. Biol Psychol.

[83] Gunner G, Cheadle L, Johnson KM, Ayata P, Badimon A, Mondo E (2019). Sensory lesioning induces microglial synapse elimination via ADAM10 and fractalkine signaling. Nat Neurosci.

[84] Hogan MK, Hamilton GF, Horner PJ (2020). Neural Stimulation and Molecular Mechanisms of Plasticity and Regeneration: A Review. Front Cell Neurosci.

[85] Ferro A, Auguste YSS, Cheadle L (2021). Microglia, Cytokines, and Neural Activity: Unexpected Interactions in Brain Development and Function. Front Immunol.

[86] Holtmaat A, De Paola V, Wilbrecht L, Knott GW (2008). Imaging of experience-dependent structural plasticity in the mouse neocortex in vivo. Behav Brain Res.

[87] Nanou E, Catterall WA (2018). Calcium Channels, Synaptic Plasticity, and Neuropsychiatric Disease. Neuron.

[88] Flamand MN, Meyer KD (2019). The epitranscriptome and synaptic plasticity. Curr Opin Neurobiol.

[89] Morrison AP, Frame L, Larkin W (2003). Relationships between trauma and psychosis: a review and integration. Br J Clin Psychol.

[90] Gawęda Ł, Göritz AS, Moritz S (2019). Mediating role of aberrant salience and self-disturbances for the relationship between childhood trauma and psychotic-like experiences in the general population. Schizophr Res.

[91] Tomassi S, Tosato S (2017). Epigenetics and gene expression profile in first-episode psychosis: The role of childhood trauma. Neurosci Biobehav Rev.

[92] Luigi M, Dellazizzo L, Giguère C-É, Goulet M-H, Dumais A. Shedding Light on “the Hole”: A Systematic Review and Meta-Analysis on Adverse Psychological Effects and Mortality Following Solitary Confinement in Correctional Settings. Front Psychiatry 2020;11. 10.3389/fpsyt.2020.00840. 10.3389/fpsyt.2020.00840 PMC7468496 32973582

[93] Huckle C, Lemmel F, Johnson S (2021). Experiences of friendships of young people with first-episode psychosis: A qualitative study. PLoS One.

[94] Dantchev S, Zammit S, Wolke D (2018). Sibling bullying in middle childhood and psychotic disorder at 18 years: a prospective cohort study. Psychol Med.

[95] Allison G, Harrop C, Ellett L (2013). Perception of peer group rank of individuals with early psychosis. Br J Clin Psychol.

[96] Bicks LK, Peng M, Taub A, Akbarian S, Morishita H (2021). An Adolescent Sensitive Period for Social Dominance Hierarchy Plasticity Is Regulated by Cortical Plasticity Modulators in Mice. Front Neural Circuits.

[97] Yamamuro K, Bicks LK, Leventhal MB, Kato D, Im S, Flanigan ME (2020). A prefrontal-paraventricular thalamus circuit requires juvenile social experience to regulate adult sociability in mice. Nat Neurosci.

[98] Hoffman RE (2007). A social deafferentation hypothesis for induction of active schizophrenia. Schizophr Bull.

[99] Tienari P (1991). Interaction between genetic vulnerability and family environment: the Finnish adoptive family study of schizophrenia. Acta Psychiatr Scand.

[100] Bjornestad J, ten Velden Hegelstad W, Joa I, Davidson L, Larsen TK, Melle I (2017). “With a little help from my friends” social predictors of clinical recovery in first-episode psychosis. Psychiatry Res.

[101] Lecomte T, Potvin S, Samson C, Francoeur A, Hache-Labelle C, Gagné S (2019). Predicting and preventing symptom onset and relapse in schizophrenia—A metareview of current empirical evidence. J Abnorm Psychol.

[102] Gaag Mvander, van der Gaag M, van den Berg D, Ising H (2019). CBT in the prevention of psychosis and other severe mental disorders in patients with an at risk mental state: A review and proposed next steps. Schizophrenia Res.

[103] Moritz S, Klein JP, Lysaker PH, Mehl S (2019). Metacognitive and cognitive-behavioral interventions for psychosis: new developments. Dialogues Clin Neurosci.

[104] Turner DT, McGlanaghy E, Cuijpers P, van der Gaag M, Karyotaki E, MacBeth A (2018). A Meta-Analysis of Social Skills Training and Related Interventions for Psychosis. Schizophr Bull.

[105] Mueser KT, Drake RE, Bond GR (2016). Recent advances in supported employment for people with serious mental illness. Curr Opin Psychiatry.

[106] Craig TK, Rus-Calafell M, Ward T, Leff JP, Huckvale M, Howarth E (2018). AVATAR therapy for auditory verbal hallucinations in people with psychosis: a single-blind, randomised controlled trial. Lancet Psychiatry.

[107] Mason L, Peters ER, Dima D, Williams SC, Kumari V (2016). Cognitive Behavioral Therapy Normalizes Functional Connectivity for Social Threat in Psychosis. Schizophr Bull.

[108] Mason L, Peters E, Williams SC, Kumari V (2017). Brain connectivity changes occurring following cognitive behavioural therapy for psychosis predict long-term recovery. Transl Psychiatry.

[109] Keshavan MS, Eack SM, Prasad KM, Haller CS, Cho RY (2017). Longitudinal functional brain imaging study in early course schizophrenia before and after cognitive enhancement therapy. Neuroimage.

[110] Guimond S, Ling G, Drodge J, Matheson H, Wojtalik JA, Lopez B, et al. Functional connectivity associated with improvement in emotion management after cognitive enhancement therapy in early-course schizophrenia. Psychol Med 2020;52:2245–54. 10.1017/S0033291720004110 PMC10763577 33183362

[111] Green MF (2016). Impact of cognitive and social cognitive impairment on functional outcomes in patients with schizophrenia. J Clin Psychiatry.

[112] Fervaha G, Siddiqui I, Foussias G, Agid O, Remington G (2015). Motivation and Social Cognition in Patients with Schizophrenia. J Int Neuropsychol Soc.

[113] Miley K, Meyer-Kalos P, Ma S, Bond DJ, Kummerfeld E, Vinogradov S. Causal pathways to social and occupational functioning in the first episode of schizophrenia: uncovering unmet treatment needs. Psychol Med. 2021;1:1–9. 10.1017/S0033291721003780 PMC10106305 37310333

[114] Nahum M, Lee H, Fisher M, Green MF, Hooker CI, Ventura J (2021). Online Social Cognition Training in Schizophrenia: A Double-Blind, Randomized, Controlled Multi-Site Clinical Trial. Schizophr Bull.

[115] Miley K, Fisher M, Nahum M, Howard E, Rowlands A, Brandrett B (2020). Six month durability of targeted cognitive training supplemented with social cognition exercises in schizophrenia. Schizophr Res Cogn.

[116] Schlosser DA, Campellone TR, Truong B, Etter K, Vergani S, Komaiko K (2018). Efficacy of PRIME, a Mobile App Intervention Designed to Improve Motivation in Young People With Schizophrenia. Schizophr Bull.

[117] Ng B. The Neuroscience of Growth Mindset and Intrinsic Motivation. Brain Sci. 2018;8. 10.3390/brainsci8020020. 10.3390/brainsci8020020 PMC5836039 29373496

[118] Hogarty GE, Flesher S (1999). Developmental theory for a cognitive enhancement therapy of schizophrenia. Schizophr Bull.

[119] McGurk SR, Mueser KT, Xie H, Welsh J, Kaiser S, Drake RE (2015). Cognitive Enhancement Treatment for People With Mental Illness Who Do Not Respond to Supported Employment: A Randomized Controlled Trial. Am J Psychiatry.

[120] Nienow TM, MacDonald AW, Lim KO (2016). TDCS produces incremental gain when combined with working memory training in patients with schizophrenia: A proof of concept pilot study. Schizophr Res.

[121] Hadar R, Bikovski L, Soto-Montenegro ML, Schimke J, Maier P, Ewing S (2018). Early neuromodulation prevents the development of brain and behavioral abnormalities in a rodent model of schizophrenia. Mol Psychiatry.

[122] Bovy L, Berkers RMWJ, Pottkämper JCM, Varatheeswaran R, Fernández G, Tendolkar I (2020). Transcranial Magnetic Stimulation of the Medial Prefrontal Cortex Decreases Emotional Memory Schemas. Cereb Cortex.

[123] Bohacek J, Farinelli M, Mirante O, Steiner G, Gapp K, Coiret G (2015). Pathological brain plasticity and cognition in the offspring of males subjected to postnatal traumatic stress. Mol Psychiatry.

[124] Reh RK, Dias BG, Nelson CA, Kaufer D, Werker JF, Kolb B (2020). Critical period regulation across multiple timescales. Proc Natl Acad Sci USA.

[125] Scheyer AF, Borsoi M, Pelissier-Alicot A-L, Manzoni OJJ (2020). Perinatal THC exposure via lactation induces lasting alterations to social behavior and prefrontal cortex function in rats at adulthood. Neuropsychopharmacology.

[126] Miller ML, Chadwick B, Dickstein DL, Purushothaman I, Egervari G, Rahman T (2019). Adolescent exposure to Δ9-tetrahydrocannabinol alters the transcriptional trajectory and dendritic architecture of prefrontal pyramidal neurons. Mol Psychiatry.

[127] Gomes FV, Zhu X, Grace AA (2019). Stress during critical periods of development and risk for schizophrenia. Schizophr Res.

[128] Baik J-H (2020). Stress and the dopaminergic reward system. Exp Mol Med.

[129] Fitzgerald PJ, Giustino TF, Seemann JR, Maren S (2015). Noradrenergic blockade stabilizes prefrontal activity and enables fear extinction under stress. Proc Natl Acad Sci USA.

[130] Cameron JL, Eagleson KL, Fox NA, Hensch TK, Levitt P (2017). Social Origins of Developmental Risk for Mental and Physical Illness. J Neurosci.

[131] Jenkins TA, Harte MK, Stenson G, Reynolds GP (2009). Neonatal lipopolysaccharide induces pathological changes in parvalbumin immunoreactivity in the hippocampus of the rat. Behav Brain Res.

[132] Brenhouse HC, Andersen SL (2011). Nonsteroidal anti-inflammatory treatment prevents delayed effects of early life stress in rats. Biol Psychiatry.

[133] Schiavone S, Sorce S, Dubois-Dauphin M, Jaquet V, Colaianna M, Zotti M (2009). Involvement of NOX2 in the development of behavioral and pathologic alterations in isolated rats. Biol Psychiatry.

[134] Venturino A, Schulz R, De Jesús-Cortés H, Maes ME, Nagy B, Reilly-Andújar F (2021). Microglia enable mature perineuronal nets disassembly upon anesthetic ketamine exposure or 60-Hz light entrainment in the healthy brain. Cell Rep.

[135] Carulli D, Verhaagen J. An Extracellular Perspective on CNS Maturation: Perineuronal Nets and the Control of Plasticity. Int J Mol Sci. 2021;22. 10.3390/ijms22052434. 10.3390/ijms22052434 PMC7957817 33670945

[136] Morishita H, Cabungcal J-H, Chen Y, Do KQ, Hensch TK (2015). Prolonged Period of Cortical Plasticity upon Redox Dysregulation in Fast-Spiking Interneurons. Biol Psychiatry.

[137] Do KQ, Cuenod M, Hensch TK (2015). Targeting Oxidative Stress and Aberrant Critical Period Plasticity in the Developmental Trajectory to Schizophrenia. Schizophr Bull.

[138] Sellgren CM, Gracias J, Watmuff B, Biag JD, Thanos JM, Whittredge PB (2019). Increased synapse elimination by microglia in schizophrenia patient-derived models of synaptic pruning. Nat Neurosci.

[139] Catale C, Gironda S, Lo Iacono L, Carola V. Microglial Function in the Effects of Early-Life Stress on Brain and Behavioral Development. J Clin Med Res. 2020;9. 10.3390/jcm9020468. 10.3390/jcm9020468 PMC7074320 32046333

[140] Picard K, St-Pierre M-K, Vecchiarelli HA, Bordeleau M, Tremblay M-È (2021). Neuroendocrine, neuroinflammatory and pathological outcomes of chronic stress: A story of microglial remodeling. Neurochem Int.

[141] Marín O (2012). Interneuron dysfunction in psychiatric disorders. Nat Rev Neurosci.

[142] Voss P, Thomas ME, Cisneros-Franco JM, de Villers-Sidani É (2017). Dynamic Brains and the Changing Rules of Neuroplasticity: Implications for Learning and Recovery. Front Psychol.

[143] Oberman L, Pascual-Leone A (2013). Changes in plasticity across the lifespan: cause of disease and target for intervention. Prog Brain Res.

[144] Caballero A, Flores-Barrera E, Thomases DR, Tseng KY (2020). Downregulation of parvalbumin expression in the prefrontal cortex during adolescence causes enduring prefrontal disinhibition in adulthood. Neuropsychopharmacology.

[145] Smith MR, Readhead B, Dudley JT, Morishita H (2019). Critical period plasticity-related transcriptional aberrations in schizophrenia and bipolar disorder. Schizophr Res.

[146] Hagihara H, Ohira K, Takao K, Miyakawa T (2014). Transcriptomic evidence for immaturity of the prefrontal cortex in patients with schizophrenia. Mol Brain.

[147] Bitanihirwe BKY, Mauney SA, Woo T-UW (2016). Weaving a Net of Neurobiological Mechanisms in Schizophrenia and Unraveling the Underlying Pathophysiology. Biol Psychiatry.

[148] Kaar SJ, Angelescu I, Marques TR, Howes OD (2019). Pre-frontal parvalbumin interneurons in schizophrenia: a meta-analysis of post-mortem studies. J Neural Transm.

[149] de Villers-Sidani E, Simpson KL, Lu Y-F, Lin RCS, Merzenich MM (2008). Manipulating critical period closure across different sectors of the primary auditory cortex. Nat Neurosci.

[150] LeBlanc JJ, Fagiolini M (2011). Autism: a “critical period” disorder?. Neural Plast.

[151] Yashiro K, Riday TT, Condon KH, Roberts AC, Bernardo DR, Prakash R (2009). Ube3a is required for experience-dependent maturation of the neocortex. Nat Neurosci.

[152] Hazlett HC, Gu H, Munsell BC, Kim SH, Styner M, Wolff JJ (2017). Early brain development in infants at high risk for autism spectrum disorder. Nature.

[153] Kochunov P, Hong LE (2014). Neurodevelopmental and neurodegenerative models of schizophrenia: white matter at the center stage. Schizophr Bull.

[154] Cetin-Karayumak S, Di Biase MA, Chunga N, Reid B, Somes N, Lyall AE (2020). White matter abnormalities across the lifespan of schizophrenia: a harmonized multi-site diffusion MRI study. Mol Psychiatry.

[155] Anticevic A, Hu X, Xiao Y, Hu J, Li F, Bi F (2015). Early-course unmedicated schizophrenia patients exhibit elevated prefrontal connectivity associated with longitudinal change. J Neurosci.

[156] Whitfield-Gabrieli S, Thermenos HW, Milanovic S, Tsuang MT, Faraone SV, McCarley RW (2009). Hyperactivity and hyperconnectivity of the default network in schizophrenia and in first-degree relatives of persons with schizophrenia. Proc Natl Acad Sci USA.

[157] Sun L, Castellanos N, Grützner C, Koethe D, Rivolta D, Wibral M (2013). Evidence for dysregulated high-frequency oscillations during sensory processing in medication-naïve, first episode schizophrenia. Schizophr Res.

[158] Uhlhaas PJ (2013). Dysconnectivity, large-scale networks and neuronal dynamics in schizophrenia. Curr Opin Neurobiol.

[159] Clementz BA, Parker DA, Trotti RL, McDowell JE, Keedy SK, Keshavan MS (2022). Psychosis Biotypes: Replication and Validation from the B-SNIP Consortium. Schizophr Bull.

[160] Clancy K, Ding M, Bernat E, Schmidt NB, Li W (2017). Restless “rest”: intrinsic sensory hyperactivity and disinhibition in post-traumatic stress disorder. Brain.

[161] Cannon TD, Chung Y, He G, Sun D, Jacobson A, van Erp TGM (2015). Progressive reduction in cortical thickness as psychosis develops: a multisite longitudinal neuroimaging study of youth at elevated clinical risk. Biol Psychiatry.

[162] Di Biase MA, Cetin-Karayumak S, Lyall AE, Zalesky A, Cho KIK, Zhang F (2021). White matter changes in psychosis risk relate to development and are not impacted by the transition to psychosis. Mol Psychiatry.

[163] Xin W, Chan JR (2020). Myelin plasticity: sculpting circuits in learning and memory. Nat Rev Neurosci.

[164] Andreasen NC, Nopoulos P, Magnotta V, Pierson R, Ziebell S, Ho B-C (2011). Progressive brain change in schizophrenia: a prospective longitudinal study of first-episode schizophrenia. Biol Psychiatry.

[165] Schnack HG, van Haren NEM, Nieuwenhuis M, Hulshoff Pol HE, Cahn W, Kahn RS (2016). Accelerated Brain Aging in Schizophrenia: A Longitudinal Pattern Recognition Study. Am J Psychiatry.

[166] Cropley VL, Klauser P, Lenroot RK, Bruggemann J, Sundram S, Bousman C (2017). Accelerated Gray and White Matter Deterioration With Age in Schizophrenia. Am J Psychiatry.

[167] Stauffer E-M, Bethlehem RAI, Warrier V, Murray GK, Romero-Garcia R, Seidlitz J (2021). Grey and white matter microstructure is associated with polygenic risk for schizophrenia. Mol Psychiatry.

[168] Walker FR, Beynon SB, Jones KA, Zhao Z, Kongsui R, Cairns M (2014). Dynamic structural remodelling of microglia in health and disease: A review of the models, the signals and the mechanisms. Brain Behav Immun.

[169] Mehta UM, Thanki MV, Padmanabhan J, Pascual-Leone A, Keshavan MS (2019). Motor cortical plasticity in schizophrenia: A meta-analysis of Transcranial Magnetic Stimulation-Electromyography studies. Schizophr Res.

[170] Hasselmo ME, Sarter M (2011). Modes and models of forebrain cholinergic neuromodulation of cognition. Neuropsychopharmacology.

[171] Shepard KN, Liles LC, Weinshenker D, Liu RC (2015). Norepinephrine is necessary for experience-dependent plasticity in the developing mouse auditory cortex. J Neurosci.

[172] Vinogradov S, Fisher M, Warm H, Holland C, Kirshner MA, Pollock BG (2009). The cognitive cost of anticholinergic burden: decreased response to cognitive training in schizophrenia. Am J Psychiatry.

[173] Joshi YB, Thomas ML, Braff DL, Green MF, Gur RC, Gur RE (2021). Anticholinergic Medication Burden-Associated Cognitive Impairment in Schizophrenia. Am J Psychiatry.

[174] Adams R, Pinotsis D, Tsirlis K, Ji JL, Repovs G, Murray J (2021). Computational Modelling of EEG and fMRI Paradigms Reveals a Consistent Loss of Pyramidal Cell Synaptic Gain in Schizophrenia. Biol Psychiatry.

[175] Adams RA, Bush D, Zheng F, Meyer SS, Kaplan R, Orfanos S (2020). Impaired theta phase coupling underlies frontotemporal dysconnectivity in schizophrenia. Brain.

[176] Giraldo-Chica M, Woodward ND (2017). Review of thalamocortical resting-state fMRI studies in schizophrenia. Schizophr Res.

[177] Anticevic A, Cole MW, Repovs G, Murray JD, Brumbaugh MS, Winkler AM (2014). Characterizing thalamo-cortical disturbances in Schizophrenia and bipolar illness. Cereb Cortex.

[178] Anticevic A, Haut K, Murray JD, Repovs G, Yang GJ, Diehl C (2015). Association of Thalamic Dysconnectivity and Conversion to Psychosis in Youth and Young Adults at Elevated Clinical Risk. JAMA Psychiatry.

[179] Benoit LJ, Holt ES, Posani L, Fusi S, Harris AZ, Canetta S (2022). Adolescent thalamic inhibition leads to long-lasting impairments in prefrontal cortex function. Nat Neurosci.

[180] Schmitt LI, Wimmer RD, Nakajima M, Happ M, Mofakham S, Halassa MM (2017). Thalamic amplification of cortical connectivity sustains attentional control. Nature.

[181] Herman AB, Brown EG, Dale CL, Hinkley LB, Subramaniam K, Houde JF (2020). The Visual Word Form Area compensates for auditory working memory dysfunction in schizophrenia. Sci Rep.

[182] Ramsay IS, Nienow TM, MacDonald AW (2017). Increases in Intrinsic Thalamocortical Connectivity and Overall Cognition Following Cognitive Remediation in Chronic Schizophrenia. Biol Psychiatry: Cogn Neurosci Neuroimaging.

[183] Jena PK, Sheng L, Di Lucente J, Jin L-W, Maezawa I, Wan Y-JY (2018). Dysregulated bile acid synthesis and dysbiosis are implicated in Western diet-induced systemic inflammation, microglial activation, and reduced neuroplasticity. FASEB J.

[184] Firth J, Stubbs B, Rosenbaum S, Vancampfort D, Malchow B, Schuch F (2017). Aerobic exercise improves cognitive functioning in people with schizophrenia: a systematic review and meta-analysis. Schizophr Bull.

[185] Fang W, Zhang J, Hong L, Huang W, Dai X, Ye Q (2020). Metformin ameliorates stress-induced depression-like behaviors via enhancing the expression of BDNF by activating AMPK/CREB-mediated histone acetylation. J Affect Disord.

[186] White-Schwoch T, Woodruff Carr K, Anderson S, Strait DL, Kraus N (2013). Older adults benefit from music training early in life: biological evidence for long-term training-driven plasticity. J Neurosci.

[187] Perkins DO, Jeffries CD, Do KQ (2020). Potential Roles of Redox Dysregulation in the Development of Schizophrenia. Biol Psychiatry.

[188] Steullet P, Cabungcal JH, Monin A, Dwir D, O’Donnell P, Cuenod M (2016). Redox dysregulation, neuroinflammation, and NMDA receptor hypofunction: A “central hub” in schizophrenia pathophysiology?. Schizophr Res.

[189] Eack SM, Hogarty GE, Cho RY, Prasad KMR, Greenwald DP, Hogarty SS (2010). Neuroprotective effects of cognitive enhancement therapy against gray matter loss in early schizophrenia: results from a 2-year randomized controlled trial. Arch Gen Psychiatry.

[190] Çakici N, van Beveren NJM, Judge-Hundal G, Koola MM, Sommer IEC (2019). An update on the efficacy of anti-inflammatory agents for patients with schizophrenia: a meta-analysis. Psychol Med.

[191] Jacob MS, Roach BJ, Hamilton HK, Carrión RE, Belger A, Duncan E (2021). Visual cortical plasticity and the risk for psychosis: An interim analysis of the North American Prodrome Longitudinal Study. Schizophr Res.

[192] Adams RA, Pinotsis D, Tsirlis K, Unruh L, Mahajan A, Horas AM (2022). Computational Modeling of Electroencephalography and Functional Magnetic Resonance Imaging Paradigms Indicates a Consistent Loss of Pyramidal Cell Synaptic Gain in Schizophrenia. Biol Psychiatry.

[193] Friston KJ, Frith CD (1995). Schizophrenia: a disconnection syndrome?. Clin Neurosci.

[194] Nazeri A, Chakravarty MM, Felsky D, Lobaugh NJ, Rajji TK, Mulsant BH (2013). Alterations of superficial white matter in schizophrenia and relationship to cognitive performance. Neuropsychopharmacology.

[195] Javitt DC, Sweet RA (2015). Auditory dysfunction in schizophrenia: integrating clinical and basic features. Nat Rev Neurosci.

[196] Allen P, Seal ML, Valli I, Fusar-Poli P, Perlini C, Day F (2011). Altered prefrontal and hippocampal function during verbal encoding and recognition in people with prodromal symptoms of psychosis. Schizophr Bull.

[197] Golia MT, Poggini S, Alboni S, Garofalo S, Ciano Albanese N, Viglione A (2019). Interplay between inflammation and neural plasticity: Both immune activation and suppression impair LTP and BDNF expression. Brain Behav Immun.

[198] Shen W, Plotkin JL, Francardo V, Ko WKD, Xie Z, Li Q (2015). M4 Muscarinic Receptor Signaling Ameliorates Striatal Plasticity Deficits in Models of L-DOPA-Induced Dyskinesia. Neuron.

[199] Zhou C, Peng B, Qin Z, Zhu W, Guo C (2021). Metformin attenuates LPS-induced neuronal injury and cognitive impairments by blocking NF-κB pathway. BMC Neurosci.

[200] Muñoz-Arenas G, Pulido G, Treviño S, Vázquez-Roque R, Flores G, Moran C (2020). Effects of metformin on recognition memory and hippocampal neuroplasticity in rats with metabolic syndrome. Synapse.

[201] Ganai SA, Ramadoss M, Mahadevan V (2016). Histone Deacetylase (HDAC) Inhibitors - emerging roles in neuronal memory, learning, synaptic plasticity and neural regeneration. Curr Neuropharmacol.

[202] Fraguas D, Díaz-Caneja CM, Ayora M, Hernández-Álvarez F, Rodríguez-Quiroga A, Recio S (2019). Oxidative Stress and Inflammation in First-Episode Psychosis: A Systematic Review and Meta-analysis. Schizophr Bull.

